# Supplementary figures and images for: A machine learning-based phenotype for long COVID in children: An EHR-based study from the RECOVER program
Source: PLoS One. 2023 Aug 10;18(8):e0289774. doi: 10.1371/journal.pone.0289774 (PMC10414557; doi:10.1371/journal.pone.0289774)

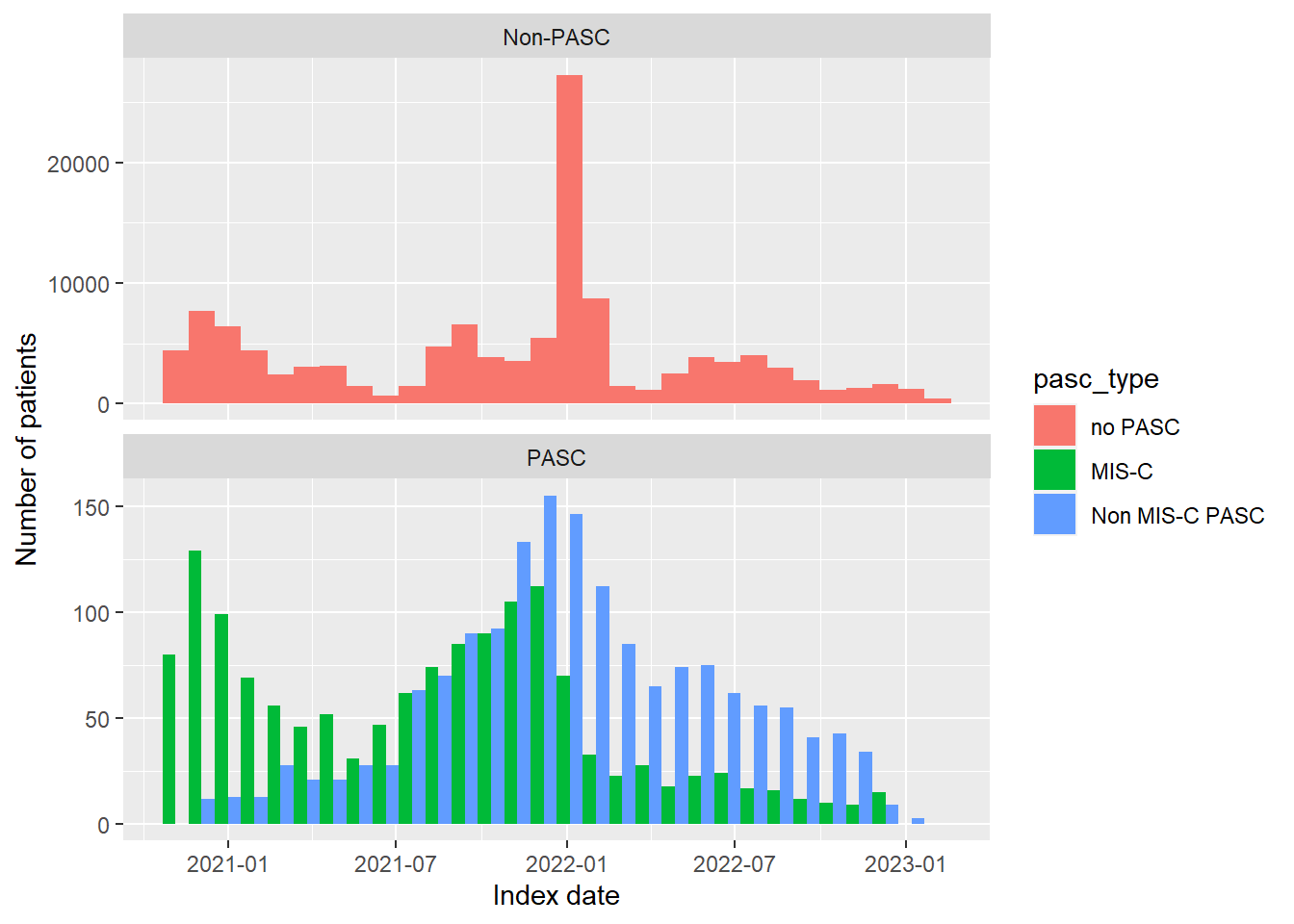

Supplement: S1 Fig — (TIF) [file pone.0289774.s001.tif]

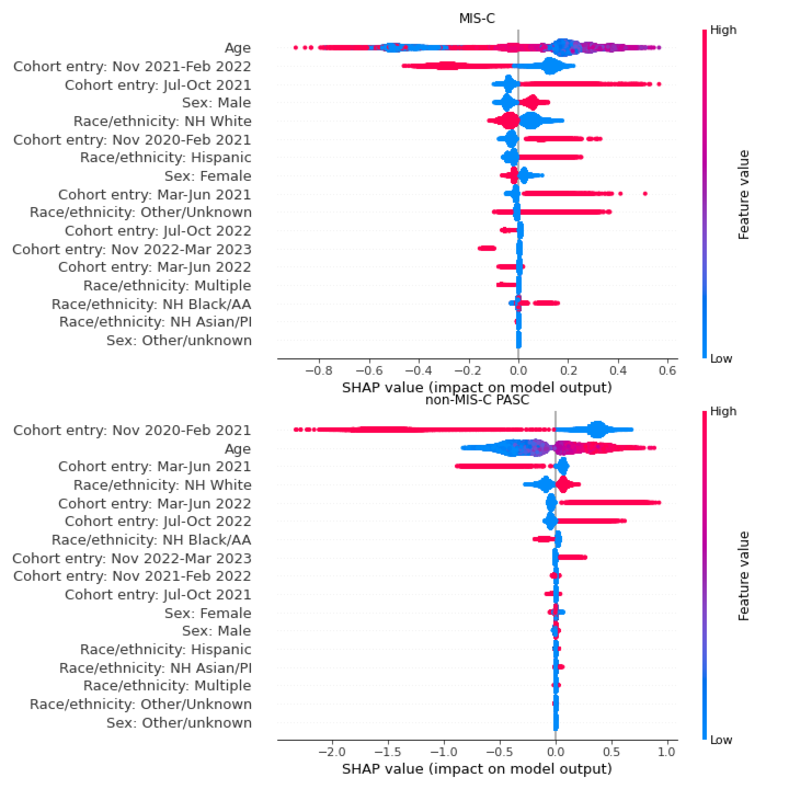

Supplement: S2 Fig — The plots show the most significant features as determined by the sum of SHAP value magnitudes over all samples. For each feature, SHAP values for each patient are plotted, with color representing the feature value (e.g. red if feature was present and blue if absent in case of a binary variable). The SHAP values pictured are for the 3 class classification task and the x axis is interpreted as change in log odds for the corresponding outcome (e.g. MIS-C) as opposed to change in probability (in particular, SHAP values are not confined to be between –1 and 1). (ZIP) [file pone.0289774.s002.zip › Supplementary Figure 2a.tif]

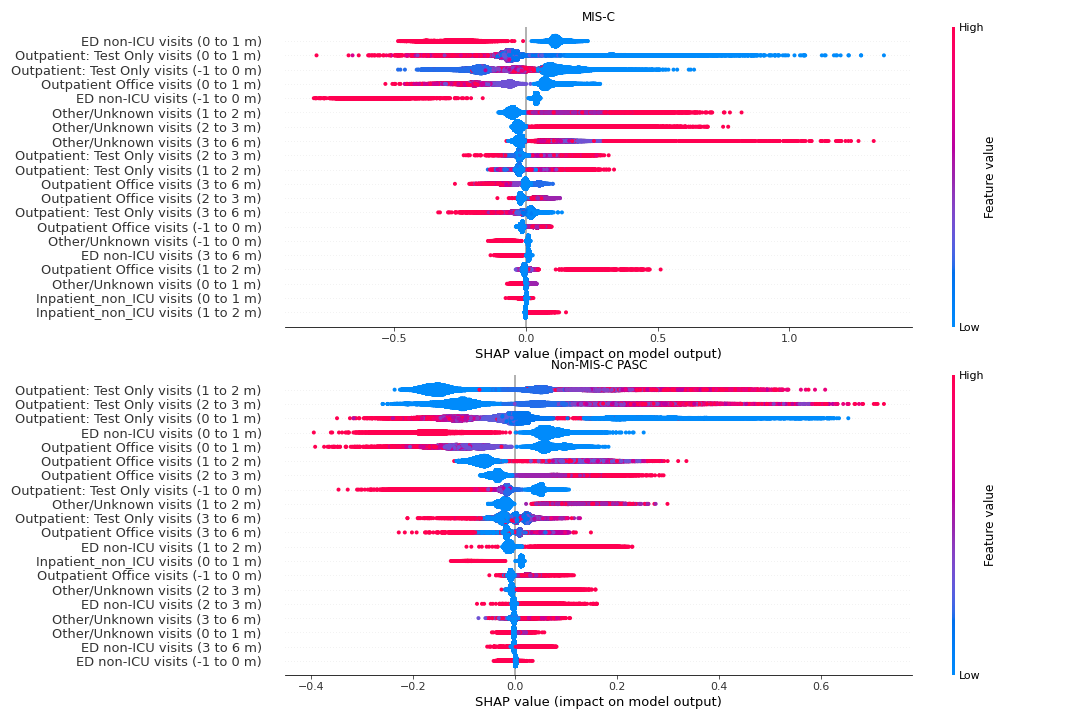

Supplement: S2 Fig — The plots show the most significant features as determined by the sum of SHAP value magnitudes over all samples. For each feature, SHAP values for each patient are plotted, with color representing the feature value (e.g. red if feature was present and blue if absent in case of a binary variable). The SHAP values pictured are for the 3 class classification task and the x axis is interpreted as change in log odds for the corresponding outcome (e.g. MIS-C) as opposed to change in probability (in particular, SHAP values are not confined to be between –1 and 1). (ZIP) [file pone.0289774.s002.zip › Supplementary Figure 2b.tif]

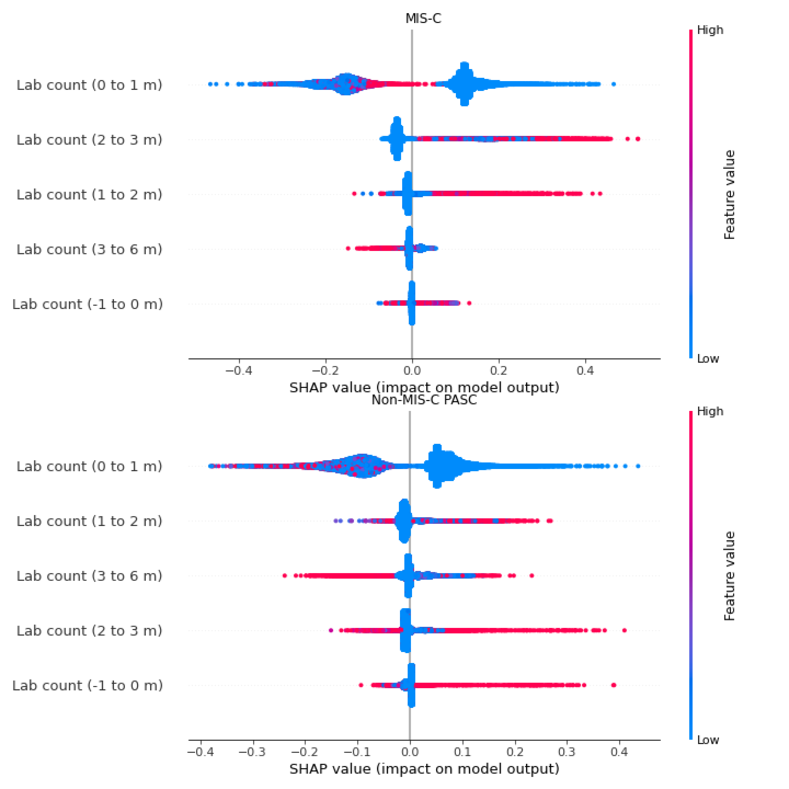

Supplement: S2 Fig — The plots show the most significant features as determined by the sum of SHAP value magnitudes over all samples. For each feature, SHAP values for each patient are plotted, with color representing the feature value (e.g. red if feature was present and blue if absent in case of a binary variable). The SHAP values pictured are for the 3 class classification task and the x axis is interpreted as change in log odds for the corresponding outcome (e.g. MIS-C) as opposed to change in probability (in particular, SHAP values are not confined to be between –1 and 1). (ZIP) [file pone.0289774.s002.zip › Supplementary Figure 2c.tif]

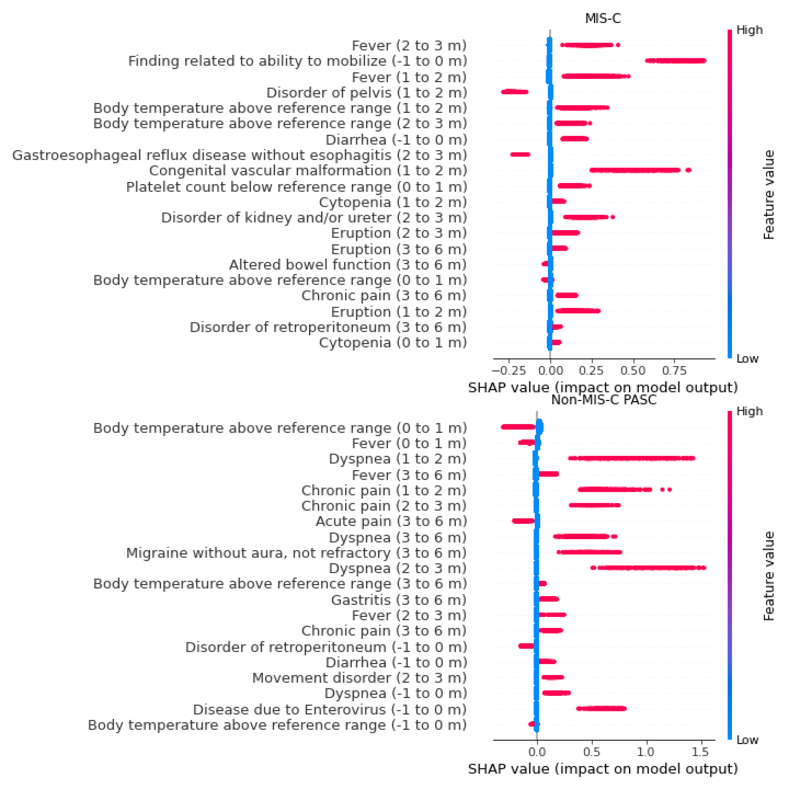

Supplement: S2 Fig — The plots show the most significant features as determined by the sum of SHAP value magnitudes over all samples. For each feature, SHAP values for each patient are plotted, with color representing the feature value (e.g. red if feature was present and blue if absent in case of a binary variable). The SHAP values pictured are for the 3 class classification task and the x axis is interpreted as change in log odds for the corresponding outcome (e.g. MIS-C) as opposed to change in probability (in particular, SHAP values are not confined to be between –1 and 1). (ZIP) [file pone.0289774.s002.zip › Supplementary Figure 2d.tif]

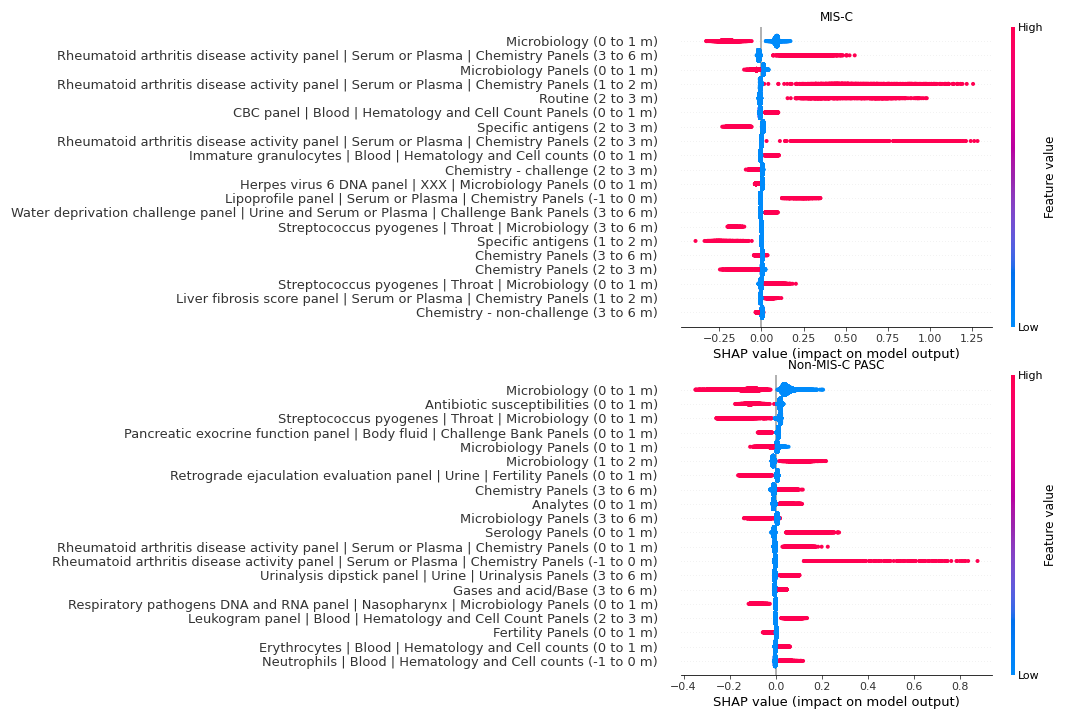

Supplement: S2 Fig — The plots show the most significant features as determined by the sum of SHAP value magnitudes over all samples. For each feature, SHAP values for each patient are plotted, with color representing the feature value (e.g. red if feature was present and blue if absent in case of a binary variable). The SHAP values pictured are for the 3 class classification task and the x axis is interpreted as change in log odds for the corresponding outcome (e.g. MIS-C) as opposed to change in probability (in particular, SHAP values are not confined to be between –1 and 1). (ZIP) [file pone.0289774.s002.zip › Supplementary Figure 2e.tif]

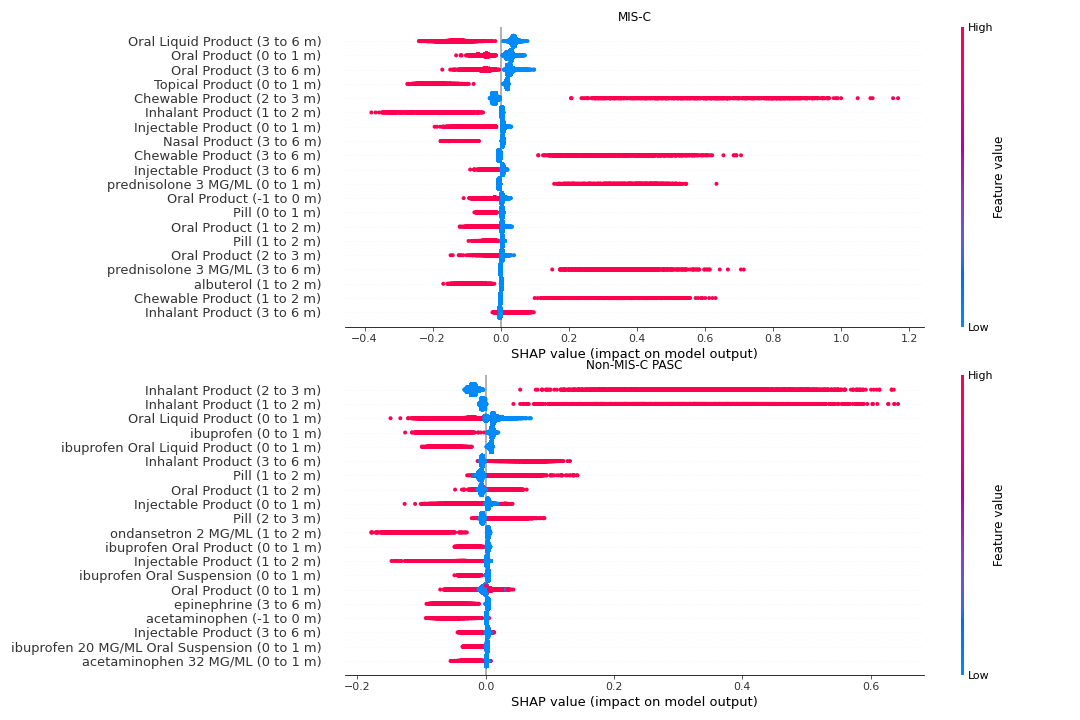

Supplement: S2 Fig — The plots show the most significant features as determined by the sum of SHAP value magnitudes over all samples. For each feature, SHAP values for each patient are plotted, with color representing the feature value (e.g. red if feature was present and blue if absent in case of a binary variable). The SHAP values pictured are for the 3 class classification task and the x axis is interpreted as change in log odds for the corresponding outcome (e.g. MIS-C) as opposed to change in probability (in particular, SHAP values are not confined to be between –1 and 1). (ZIP) [file pone.0289774.s002.zip › Supplementary Figure 2f.tif]

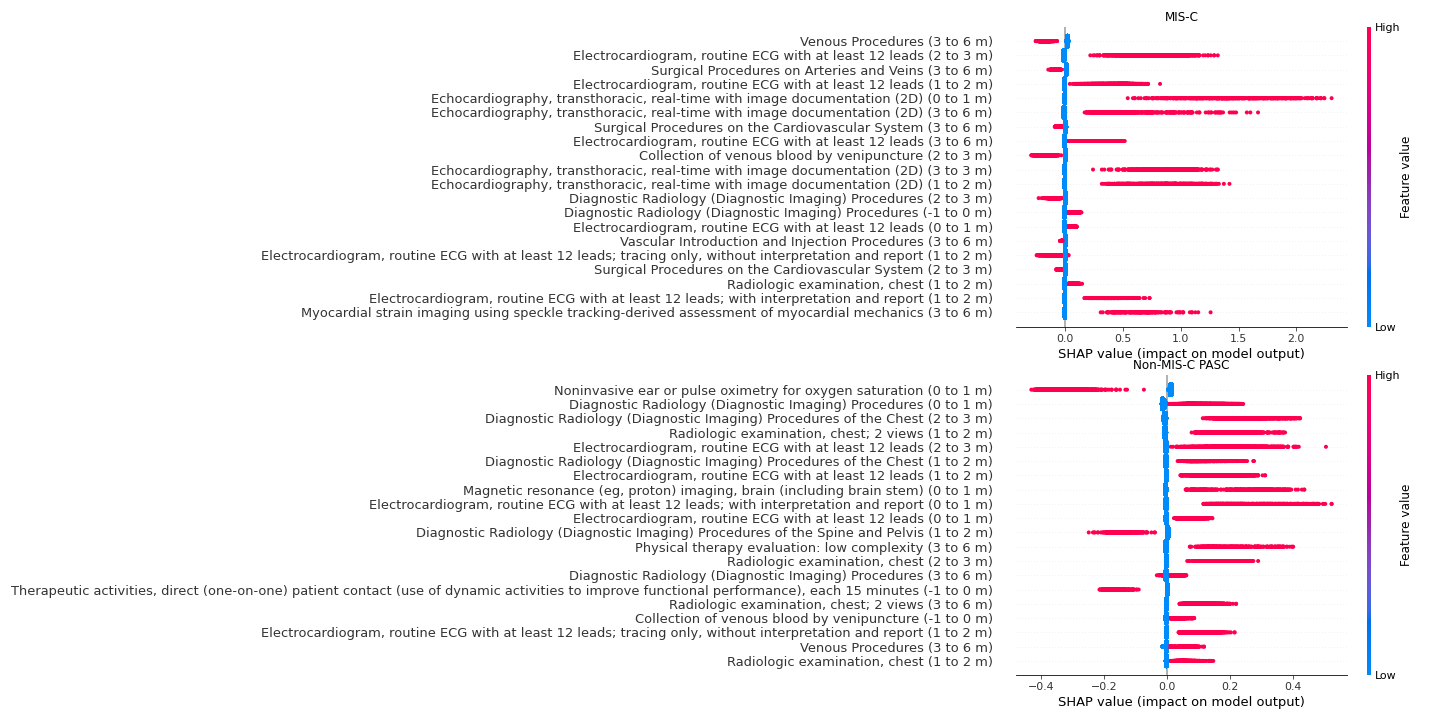

Supplement: S2 Fig — The plots show the most significant features as determined by the sum of SHAP value magnitudes over all samples. For each feature, SHAP values for each patient are plotted, with color representing the feature value (e.g. red if feature was present and blue if absent in case of a binary variable). The SHAP values pictured are for the 3 class classification task and the x axis is interpreted as change in log odds for the corresponding outcome (e.g. MIS-C) as opposed to change in probability (in particular, SHAP values are not confined to be between –1 and 1). (ZIP) [file pone.0289774.s002.zip › Supplementary Figure 2g.tif]
