# Supplementary material for: A machine learning-based phenotype for long COVID in children: An EHR-based study from the RECOVER program
Source: PLoS One. 2023 Aug 10;18(8):e0289774. doi: 10.1371/journal.pone.0289774 (PMC10414557; doi:10.1371/journal.pone.0289774)
Supplement: S1 Table — The table below displays several performance metrics for the XGBoost model, a random forest model, and binary logistic regression model computed using 5-fold cross-validation with the same set of features for each model. In all three models, the outcome predicted is PASC (any). Model parameters, selected by cross-validated grid search are displayed below each model. Any parameters not listed were set to the default values in the corresponding Python libraries. (DOCX) [file pone.0289774.s003.docx]

|  | XGBoost | Random Forest | Regularized logistic regression |
| --- | --- | --- | --- |
| Model parameters | n_estimators=2900,  learning_rate=0.01  colsample_bytree=0.6,  max_depth=5,  min_child_weight=2,  gamma=0,  subsample=0.8 | n_estimators=1800 | penalty: ‘l1’,  C=0.01,  solver=’saga’ |
| Accuracy | 0.990 | 0.987 | 0.986 |
| AUPR | 0.852 | 0.781 | 0.758 |
| AUROC | 0.984 | 0.977 | 0.971 |
| F1 score | 0.771 | 0.642 | 0.647 |
| Precision | 0.910 | 0.963 | 0.882 |
| Recall | 0.669 | 0.481 | 0.511 |
