# Supplementary material for: A machine learning-based phenotype for long COVID in children: An EHR-based study from the RECOVER program
Source: PLoS One. 2023 Aug 10;18(8):e0289774. doi: 10.1371/journal.pone.0289774 (PMC10414557; doi:10.1371/journal.pone.0289774)
Supplement: S2 Table — These tables show the TreeScan-selected cuts for conditions, labs, procedures, and medications. Each row describes the top node which characterizes the cluster. In other words, the node, together with all descendant codes, defines the feature cluster. (ZIP) [file pone.0289774.s004.zip › Supplementary Table 2a.docx]

| Concept | Tree Level | Log Likelihood Ratio | P value | SNOMED code |
| --- | --- | --- | --- | --- |
| Cytopenia | 6 | 1,438.40 | 0.001 | 50820005 |
| Dyspnea | 6 | 1,109.32 | 0.001 | 267036007 |
| Hypo-osmolality and or hyponatremia | 6 | 1,034.13 | 0.001 | 267447008 |
| Platelet count below reference range | 6 | 658.23 | 0.001 | 415116008 |
| Disorder of retroperitoneum | 6 | 651.17 | 0.001 | 734045002 |
| Disorder of kidney and/or ureter | 6 | 571.70 | 0.001 | 443820000 |
| Lymphocyte count abnormal | 6 | 552.72 | 0.001 | 165534000 |
| Non-rheumatic heart valve disorder | 6 | 533.65 | 0.001 | 274097009 |
| Acute renal failure syndrome | 6 | 531.53 | 0.001 | 14669001 |
| RBC count low | 6 | 496.40 | 0.001 | 165423001 |
| Decreased blood leukocyte number | 6 | 445.15 | 0.001 | 419188005 |
| Enzyme level - finding | 6 | 417.18 | 0.001 | 365767001 |
| Pleural effusion | 6 | 360.85 | 0.001 | 60046008 |
| Conduction disorder of the heart | 6 | 341.47 | 0.001 | 44808001 |
| Dehydration | 6 | 330.34 | 0.001 | 34095006 |
| Chronic pain | 6 | 308.16 | 0.001 | 82423001 |
| Globulin level - finding | 6 | 307.53 | 0.001 | 365805001 |
| Mitral valve disorder | 6 | 302.51 | 0.001 | 11851006 |
| Injury of urinary organ | 6 | 292.08 | 0.001 | 733386005 |
| Abdominal cavity injury | 6 | 258.07 | 0.001 | 284005003 |
| Potassium disorder | 6 | 230.04 | 0.001 | 24529006 |
| Increased blood leukocyte number | 6 | 228.73 | 0.001 | 414478003 |
| Hypokalemia | 7 | 222.39 | 0.001 | 43339004 |
| Tricuspid valve disorder | 6 | 220.71 | 0.001 | 20721001 |
| Tricuspid valve lesion | 6 | 195.54 | 0.001 | 301109008 |
| Liver enzyme levels - finding | 7 | 180.68 | 0.001 | 365769003 |
| Elevated liver enzymes level | 6 | 179.56 | 0.001 | 707724006 |
| Pneumonia | 6 | 170.66 | 0.001 | 233604007 |
| Infective pneumonia | 6 | 154.83 | 0.001 | 312342009 |
| Altered bowel function | 6 | 153.32 | 0.001 | 88111009 |
| Body temperature above reference range | 6 | 143.23 | 0.001 | 50177009 |
| Fever | 7 | 142.01 | 0.001 | 386661006 |
| Disturbance of consciousness | 7 | 131.69 | 0.001 | 3006004 |
| Level of consciousness - finding | 6 | 131.03 | 0.001 | 365931003 |
| Cardiomegaly | 6 | 97.00 | 0.001 | 8186001 |
| Diarrhea | 7 | 96.01 | 0.001 | 62315008 |
| Venous thrombosis | 6 | 87.51 | 0.001 | 111293003 |
| Eruption | 6 | 86.83 | 0.001 | 271807003 |
| Disease due to Gram-positive coccus | 6 | 86.27 | 0.001 | 408637006 |
| Loss of consciousness | 9 | 84.98 | 0.001 | 419045004 |
| Decreased level of consciousness | 8 | 84.92 | 0.001 | 443371007 |
| Disease due to Gram-negative bacillus | 6 | 84.77 | 0.001 | 408638001 |
| Disorder of phosphorus metabolism | 6 | 83.91 | 0.001 | 87049008 |
| Viral pneumonia | 6 | 80.58 | 0.001 | 75570004 |
| Hypercapnic respiratory failure | 6 | 68.92 | 0.001 | 709109004 |
| Infection due to Enterobacteriaceae | 7 | 65.87 | 0.001 | 128945009 |
| Disorder of pelvis | 6 | 64.88 | 0.001 | 609620004 |
| Disorder of pulmonary circulation | 6 | 58.17 | 0.001 | 39785005 |
| Impaired mobility | 7 | 55.13 | 0.001 | 82971005 |
| Pneumonia caused by Human coronavirus | 7 | 55.00 | 0.001 | 713084008 |
| Finding related to ability to mobilize | 6 | 54.50 | 0.001 | 365092005 |
| Hypoxemic respiratory failure | 6 | 50.14 | 0.001 | 10676831000119101 |
| Disease of non-coronary systemic artery | 6 | 48.61 | 0.001 | 473449006 |
| Streptococcal infectious disease | 7 | 48.51 | 0.001 | 85769006 |
| Pneumonia caused by SARS-CoV-2 | 7 | 46.76 | 0.001 | 882784691000119100 |
| Reduced mobility | 8 | 46.31 | 0.001 | 8510008 |
| Adrenal cortical hypofunction | 6 | 45.63 | 0.001 | 386584007 |
| Refractory migraine | 6 | 44.49 | 0.001 | 423894005 |
| Lower respiratory infection caused by SARS-CoV-2 | 6 | 44.17 | 0.001 | 880529761000119102 |
| Migraine without aura, not refractory | 6 | 43.57 | 0.001 | 425007008 |
| Chronic respiratory failure | 6 | 43.57 | 0.001 | 39871006 |
| Cardiomyopathy | 6 | 41.31 | 0.001 | 85898001 |
| Deficiency of micronutrients | 6 | 40.83 | 0.001 | 238111008 |
| Aortic valve disorder | 6 | 39.38 | 0.001 | 8722008 |
| Staphylococcal infectious disease | 7 | 39.11 | 0.001 | 56038003 |
| Disorder of large intestine | 6 | 37.28 | 0.001 | 119523007 |
| Abnormality of systemic vein | 6 | 36.54 | 0.001 | 448157006 |
| Supraventricular arrhythmia | 6 | 36.42 | 0.001 | 72654001 |
| Pulmonary valve lesion | 6 | 36.37 | 0.001 | 301105002 |
| Pulmonary valve disorder | 6 | 35.92 | 0.001 | 76267008 |
| Hyperglycemia | 6 | 35.64 | 0.001 | 80394007 |
| Abnormality of pulmonary valve | 6 | 35.20 | 0.001 | 448643005 |
| Increased lipid | 6 | 33.75 | 0.001 | 124042003 |
| Abnormality of aortic valve | 6 | 33.43 | 0.001 | 448743001 |
| Phagocytic cell defect | 6 | 33.43 | 0.001 | 234573000 |
| Pain in bilateral legs | 6 | 31.64 | 0.001 | 15634511000119108 |
| Hyperlipidemia | 6 | 29.69 | 0.001 | 55822004 |
| Bacterial pneumonia | 6 | 29.51 | 0.001 | 53084003 |
| Disease due to Enterovirus | 6 | 29.11 | 0.001 | 53648006 |
| Lower urinary tract finding | 7 | 27.65 | 0.001 | 106100005 |
| Deficiency of macronutrients | 6 | 26.93 | 0.001 | 238107002 |
| Vitamin D deficiency | 6 | 26.24 | 0.001 | 34713006 |
| Finding of urinary tract proper | 6 | 25.26 | 0.001 | 249273002 |
| Abnormality of atrial septum | 6 | 25.25 | 0.001 | 253363004 |
| Disorder of renal parenchyma | 6 | 24.93 | 0.001 | 767094002 |
| Neutrophil count abnormal | 6 | 24.32 | 0.001 | 165519006 |
| Congenital anomaly of thorax | 6 | 24.29 | 0.001 | 363035006 |
| Disorder of bladder | 6 | 23.19 | 0.001 | 42643001 |
| Acute pain | 6 | 22.65 | 0.001 | 274663001 |
| Gastroesophageal reflux disease without esophagitis | 6 | 21.62 | 0.001 | 266435005 |
| Atrial septal defect | 7 | 21.30 | 0.001 | 70142008 |
| Uncomplicated moderate persistent asthma | 6 | 21.04 | 0.001 | 707512002 |
| Cardiac septal defects | 6 | 20.67 | 0.001 | 253273004 |
| Gastritis | 6 | 20.38 | 0.001 | 4556007 |
| Moderate persistent asthma | 6 | 18.43 | 0.001 | 427295004 |
| Functional disorder of intestine | 6 | 18.17 | 0.001 | 81120009 |
| Uncomplicated mild persistent asthma | 6 | 18.05 | 0.001 | 707511009 |
| Micturition finding | 8 | 17.24 | 0.001 | 252041008 |
| Congenital heart disease | 6 | 16.76 | 0.001 | 13213009 |
| Disorder of female genital organs | 7 | 14.42 | 0.001 | 244938009 |
| Disorder of the lower urinary tract | 7 | 14.40 | 0.001 | 7793005 |
| Mild persistent asthma | 6 | 14.11 | 0.001 | 426979002 |
| Inflammation of large intestine | 6 | 13.61 | 0.001 | 302168000 |
| Movement disorder | 6 | 13.04 | 0.001 | 60342002 |
| Disorder of uterus | 6 | 13.03 | 0.001 | 12337004 |
| Streptococcal sore throat | 6 | 13.01 | 0.001 | 43878008 |
| Disorder of female reproductive system | 6 | 12.90 | 0.001 | 363124003 |
| Congenital vascular malformation | 6 | 12.34 | 0.001 | 400159008 |
| Disorder of colon | 7 | 12.07 | 0.002 | 128524007 |
| Finding of colon | 6 | 12.05 | 0.002 | 300310003 |
| Arterial malformation | 6 | 11.73 | 0.003 | 234119001 |
| Dysphagia | 6 | 11.59 | 0.003 | 40739000 |
| Disorder of urinary tract | 6 | 11.27 | 0.003 | 41368006 |
| Bacterial upper respiratory infection | 6 | 10.15 | 0.003 | 312118003 |
