# Supplementary material for: A machine learning-based phenotype for long COVID in children: An EHR-based study from the RECOVER program
Source: PLoS One. 2023 Aug 10;18(8):e0289774. doi: 10.1371/journal.pone.0289774 (PMC10414557; doi:10.1371/journal.pone.0289774)
Supplement: S2 Table — These tables show the TreeScan-selected cuts for conditions, labs, procedures, and medications. Each row describes the top node which characterizes the cluster. In other words, the node, together with all descendant codes, defines the feature cluster. (ZIP) [file pone.0289774.s004.zip › Supplementary Table 2b.docx]

| Concept | Tree Level | Log Likelihood Ratio | P value | LOINC code |
| --- | --- | --- | --- | --- |
| Chemistry - non-challenge | 4 | 54,718.46 | 0.001 | LP7786-9 |
| Chemistry - challenge | 4 | 43,021.76 | 0.001 | LP7784-4 |
| Chemistry Panels | 5 | 39,917.05 | 0.001 | LP7834-7 |
| Hematology and Cell Count Panels | 4 | 27,295.41 | 0.001 | LP7833-9 |
| Female fertility | 4 | 25,079.51 | 0.001 | LP32818-4 |
| NEI eyeGENE slit lamp biomicroscopy | 4 | 23,408.01 | 0.001 | LP203653-3 |
| Cell types | 5 | 23,179.35 | 0.001 | LP32763-2 |
| Male fertility | 4 | 23,179.35 | 0.001 | LP32817-6 |
| CBC W Reflex Manual Differential panel \| Blood \| Hematology and Cell Count Panels | 5 | 22,661.46 | 0.001 | LP393882-8 |
| Analytes | 4 | 18,892.73 | 0.001 | LP40317-7 |
| Other markers | 4 | 18,756.70 | 0.001 | LP32943-0 |
| Comprehensive metabolic 2000 panel \| Serum or Plasma \| Chemistry Panels | 6 | 18,463.54 | 0.001 | LP386863-7 |
| Hemogram without Platelets and with Manual Differential panel \| Blood \| Hematology and Cell Count Panels | 5 | 15,784.19 | 0.001 | LP393926-3 |
| Metabolic panel.large animal \| Serum or Plasma \| Chemistry Panels | 6 | 14,624.40 | 0.001 | LP386983-3 |
| Metabolic panel.small animal \| Serum or Plasma \| Chemistry Panels | 6 | 14,584.73 | 0.001 | LP386984-1 |
| Comprehensive metabolic 1998 panel \| Serum or Plasma \| Chemistry Panels | 6 | 14,573.57 | 0.001 | LP386862-9 |
| CBC W Differential panel, method unspecified \| Blood \| Hematology and Cell Count Panels | 5 | 13,244.33 | 0.001 | LP393880-2 |
| Manual Differential panel \| Blood \| Hematology and Cell Count Panels | 5 | 13,232.87 | 0.001 | LP393933-9 |
| Leukogram panel \| Blood \| Hematology and Cell Count Panels | 5 | 13,232.87 | 0.001 | LP393932-1 |
| Microbiology | 4 | 12,638.79 | 0.001 | LP7819-8 |
| Renal function 2000 panel \| Serum or Plasma \| Chemistry Panels | 6 | 11,721.83 | 0.001 | LP387047-6 |
| Basic metabolic and albumin panel \| Serum or Plasma \| Chemistry Panels | 6 | 11,455.32 | 0.001 | LP386816-5 |
| Microbiology Panels | 5 | 11,016.46 | 0.001 | LP7835-4 |
| Basic metabolic 2000 panel \| Serum or Plasma \| Chemistry Panels | 6 | 10,159.53 | 0.001 | LP386818-1 |
| CBC W Auto Differential panel \| Blood \| Hematology and Cell Count Panels | 5 | 10,014.93 | 0.001 | LP393878-6 |
| Metabolic panel.dialysis patient \| Serum or Plasma \| Chemistry Panels | 6 | 9,946.79 | 0.001 | LP386982-5 |
| Differential panel, method unspecified \| Blood \| Hematology and Cell Count Panels | 5 | 9,885.24 | 0.001 | LP393917-2 |
| Urinalysis Panels | 4 | 9,608.60 | 0.001 | LP7838-8 |
| Short blood count panel \| Blood \| Hematology and Cell Count Panels | 5 | 9,361.78 | 0.001 | LP393938-8 |
| Basic metabolic 2008 panel with ionized calcium \| Serum or Plasma \| Chemistry Panels | 6 | 8,988.40 | 0.001 | LP386819-9 |
| Basic metabolic 1998 panel \| Serum or Plasma \| Chemistry Panels | 6 | 8,914.69 | 0.001 | LP386817-3 |
| Routine | 4 | 8,794.66 | 0.001 | LP31624-7 |
| Urinalysis complete W Reflex Culture panel \| Urine \| Urinalysis Panels | 5 | 8,110.23 | 0.001 | LP402547-6 |
| Urinalysis complete panel \| Urine \| Urinalysis Panels | 5 | 7,908.15 | 0.001 | LP402546-8 |
| Urinalysis dipstick W Reflex Microscopic panel \| Urine \| Urinalysis Panels | 5 | 7,908.15 | 0.001 | LP402550-0 |
| Hepatic function 2000 panel \| Serum or Plasma \| Chemistry Panels | 6 | 7,871.47 | 0.001 | LP386948-6 |
| Coagulation Panels | 4 | 7,829.89 | 0.001 | LP31893-8 |
| Intravascular coagulation and fibrinolysis panel \| Patient \| Coagulation Panels | 5 | 7,629.45 | 0.001 | LP428844-7 |
| Gases and acid/Base | 5 | 7,193.56 | 0.001 | LP31400-2 |
| Challenge Bank Panels | 5 | 7,062.52 | 0.001 | LP31895-3 |
| CBC panel \| Blood \| Hematology and Cell Count Panels | 5 | 6,932.42 | 0.001 | LP393905-7 |
| Hepatic function 1996 panel \| Serum or Plasma \| Chemistry Panels | 6 | 6,606.59 | 0.001 | LP386947-8 |
| Water deprivation challenge panel \| Urine and Serum or Plasma \| Challenge Bank Panels | 6 | 6,438.75 | 0.001 | LP417169-2 |
| Vasopressin challenge post water deprivation panel \| Urine and Serum or Plasma \| Challenge Bank Panels | 6 | 6,438.75 | 0.001 | LP417168-4 |
| PT and aPTT and Fibrinogen panel \| Platelet poor plasma \| Coagulation Panels | 5 | 6,023.33 | 0.001 | LP394456-0 |
| Urinalysis dipstick W Reflex Culture panel \| Urine \| Urinalysis Panels | 5 | 5,559.58 | 0.001 | LP402549-2 |
| Electrolytes 1998 and Venous pH panel \| Serum or Plasma + Blood venous \| Chemistry Panels | 6 | 5,558.25 | 0.001 | LP386892-6 |
| Urinalysis macro (dipstick) panel \| Urine \| Urinalysis Panels | 5 | 5,353.89 | 0.001 | LP402551-8 |
| Electrolytes 1998 panel \| Serum or Plasma \| Chemistry Panels | 6 | 5,178.42 | 0.001 | LP386893-4 |
| Liver fibrosis score panel \| Serum or Plasma \| Chemistry Panels | 6 | 4,818.99 | 0.001 | LP386977-5 |
| Blood Indices | 4 | 4,509.81 | 0.001 | LP30866-5 |
| Red cell indices | 5 | 4,330.67 | 0.001 | LP31669-2 |
| PT and aPTT panel \| Platelet poor plasma \| Coagulation Panels | 5 | 4,216.85 | 0.001 | LP394457-8 |
| Smear morphology panel \| Blood \| Hematology and Cell Count Panels | 5 | 3,703.16 | 0.001 | LP393939-6 |
| Sugars/Sugar metabolism | 5 | 3,238.35 | 0.001 | LP31399-6 |
| Hemogram without Platelets panel \| Blood \| Hematology and Cell Count Panels | 5 | 3,198.76 | 0.001 | LP393927-1 |
| PT panel \| Platelet poor plasma \| Coagulation Panels | 5 | 3,182.51 | 0.001 | LP394458-6 |
| Warfarin tracking panel \| Platelet poor plasma \| Coagulation Panels | 5 | 3,182.51 | 0.001 | LP394464-4 |
| Gas panel \| Blood venous \| Chemistry Panels | 6 | 3,152.77 | 0.001 | LP386940-3 |
| Auto Differential panel \| Blood \| Hematology and Cell Count Panels | 5 | 3,095.38 | 0.001 | LP393877-8 |
| Gas and Carbon monoxide panel \| Blood venous \| Chemistry Panels | 6 | 3,087.40 | 0.001 | LP386932-0 |
| Sequential Organ Failure Assessment \| Patient \| Clinical Risk Panel | 4 | 3,070.08 | 0.001 | LP428913-0 |
| Sequential Organ Failure Assessment SOFA | 5 | 3,070.08 | 0.001 | 96789-3 |
| Urinalysis microscopic panel \| Urine sediment \| Urinalysis Panels | 5 | 2,942.37 | 0.001 | LP402554-2 |
| Mineral, bone, joint, connective tissue | 5 | 2,901.08 | 0.001 | LP31413-5 |
| Liver fibrosis score panel \| Patient \| Chemistry Panels | 6 | 2,843.21 | 0.001 | LP428840-5 |
| Nonalcoholic steatohepatitis and fibrosis panel \| Serum or Plasma \| Chemistry Panels | 6 | 2,763.94 | 0.001 | LP417161-9 |
| Erythrocyte morphology panel \| Blood \| Hematology and Cell Count Panels | 5 | 2,686.28 | 0.001 | LP393918-0 |
| Protein fractions 3 panel \| Serum or Plasma \| Chemistry Panels | 6 | 2,659.84 | 0.001 | LP387030-2 |
| EKG measurements | 4 | 2,642.83 | 0.001 | LP7795-0 |
| Chemistry - routine challenge | 4 | 2,428.77 | 0.001 | LP234174-3 |
| Protein electrophoresis and Immunoglobulins panel \| Serum \| Chemistry Panels | 6 | 2,411.00 | 0.001 | LP387029-4 |
| Lymphocytes \| Blood \| Hematology and Cell counts | 4 | 2,405.64 | 0.001 | LP392919-9 |
| Gas panel \| Blood \| Chemistry Panels | 6 | 2,399.02 | 0.001 | LP386934-6 |
| Gas and Carbon monoxide panel \| Blood \| Chemistry Panels | 6 | 2,379.92 | 0.001 | LP386929-6 |
| Basophils \| Blood \| Hematology and Cell counts | 4 | 2,375.79 | 0.001 | LP392736-7 |
| Rheumatoid arthritis disease activity panel \| Serum or Plasma \| Chemistry Panels | 6 | 2,372.29 | 0.001 | LP387049-2 |
| Cellmarker Panels | 4 | 2,372.08 | 0.001 | LP36900-6 |
| Monocytes \| Blood \| Hematology and Cell counts | 4 | 2,353.09 | 0.001 | LP393028-8 |
| Eosinophils \| Blood \| Hematology and Cell counts | 4 | 2,319.32 | 0.001 | LP392778-9 |
| Sodium and Potassium panel \| Serum or Plasma \| Chemistry Panels | 6 | 2,235.30 | 0.001 | LP387058-3 |
| Respiratory pathogens DNA and RNA 12b panel \| XXX \| Microbiology Panels | 6 | 2,147.87 | 0.001 | LP380080-4 |
| Respiratory pathogens DNA and RNA panel \| Nasopharynx \| Microbiology Panels | 6 | 2,088.62 | 0.001 | LP380083-8 |
| Protein electrophoresis panel \| Serum or Plasma \| Chemistry Panels | 6 | 2,087.86 | 0.001 | LP387033-6 |
| Immunoelectrophoresis panel \| Serum \| Chemistry Panels | 6 | 2,087.86 | 0.001 | LP386957-7 |
| Respiratory pathogens DNA and RNA 12a panel \| XXX \| Microbiology Panels | 6 | 2,078.81 | 0.001 | LP380079-6 |
| Urinalysis panel \| Urine \| Urinalysis Panels | 5 | 2,063.59 | 0.001 | LP402556-7 |
| Albumin \| Serum or Plasma \| Chemistry - non-challenge | 4 | 1,811.13 | 0.001 | LP384485-1 |
| Erythrogram panel \| Blood \| Hematology and Cell Count Panels | 5 | 1,802.70 | 0.001 | LP393919-8 |
| Immature granulocytes \| Blood \| Hematology and Cell counts | 4 | 1,793.28 | 0.001 | LP392634-4 |
| T-cell subsets CD4 and CD8 panel \| Blood \| Cellmarker Panels | 5 | 1,749.78 | 0.001 | LP400872-0 |
| Human coronavirus RNA panel \| XXX \| Microbiology Panels | 6 | 1,713.80 | 0.001 | LP380015-0 |
| Urinalysis dipstick panel \| Urine \| Urinalysis Panels | 5 | 1,688.27 | 0.001 | LP402548-4 |
| Hepatitis C virus FibroSURE panel \| Serum or Plasma \| Chemistry Panels | 6 | 1,671.03 | 0.001 | LP386949-4 |
| Hemoglobin and Hematocrit panel \| Blood \| Hematology and Cell Count Panels | 5 | 1,602.12 | 0.001 | LP393920-6 |
| Immunodeficiency follow-up panel \| XXX \| Cellmarker Panels | 5 | 1,554.17 | 0.001 | LP400855-5 |
| Amylase and Creatinine clearance panel \| Urine and Serum or Plasma \| Chemistry Panels | 6 | 1,551.77 | 0.001 | LP386810-8 |
| Bilirubin direct and total panel \| Serum or Plasma \| Chemistry Panels | 6 | 1,534.96 | 0.001 | LP386833-0 |
| Calcium-phosphorus product panel \| Serum or Plasma \| Chemistry Panels | 6 | 1,500.61 | 0.001 | LP386841-3 |
| Neutrophils \| Blood \| Hematology and Cell counts | 4 | 1,462.18 | 0.001 | LP392641-9 |
| Urea nitrogen \| Serum or Plasma \| Chemistry - non-challenge | 5 | 1,435.04 | 0.001 | LP385464-5 |
| Miscellaneous Hematology | 4 | 1,430.14 | 0.001 | LP30865-7 |
| Multiple sclerosis panel \| Serum and CSF \| Chemistry Panels | 6 | 1,426.99 | 0.001 | LP386996-5 |
| Basic metabolic panel \| Blood \| Chemistry Panels | 6 | 1,388.50 | 0.001 | LP386820-7 |
| Platelets panel \| Blood \| Hematology and Cell Count Panels | 5 | 1,371.47 | 0.001 | LP393936-2 |
| Creatinine renal clearance adjusted for body surface area panel \| Urine and Serum or Plasma \| Chemistry Panels | 6 | 1,365.43 | 0.001 | LP386873-6 |
| Cells panel \| Urine sediment \| Urinalysis Panels | 5 | 1,336.26 | 0.001 | LP402542-7 |
| Creatinine renal clearance panel \| Urine and Serum or Plasma \| Chemistry Panels | 6 | 1,325.57 | 0.001 | LP386874-4 |
| Alkaline phosphatase isoenz panel \| Serum or Plasma \| Chemistry Panels | 6 | 1,291.18 | 0.001 | LP386802-5 |
| Alkaline phosphatase \| Serum or Plasma \| Chemistry - non-challenge | 5 | 1,290.83 | 0.001 | LP382722-9 |
| Protein electrophoresis and M protein isotype panel \| Serum or Plasma \| Chemistry Panels | 6 | 1,268.82 | 0.001 | LP387028-6 |
| Monoclonal gammopathy panel \| Serum or Plasma \| Chemistry Panels | 6 | 1,268.55 | 0.001 | LP386994-0 |
| Protein \| Serum or Plasma \| Chemistry - non-challenge | 5 | 1,265.44 | 0.001 | LP384468-7 |
| Alanine aminotransferase \| Serum or Plasma \| Chemistry - non-challenge | 5 | 1,258.14 | 0.001 | LP382703-9 |
| Serology Panels | 4 | 1,247.65 | 0.001 | LP7837-0 |
| Creatinine and Glomerular filtration rate.predicted panel \| Serum, Plasma or Blood \| Chemistry Panels | 6 | 1,246.06 | 0.001 | LP386871-0 |
| Aspartate aminotransferase \| Serum or Plasma \| Chemistry - non-challenge | 5 | 1,230.53 | 0.001 | LP382836-7 |
| Carbon dioxide \| Serum or Plasma \| Chemistry - non-challenge | 4 | 1,227.94 | 0.001 | LP383334-2 |
| Parathyrin.intact and Calcium panel \| Serum or Plasma \| Chemistry Panels | 6 | 1,212.95 | 0.001 | LP387010-4 |
| Parathyrin.mid molecule and Calcium panel \| Serum or Plasma \| Chemistry Panels | 6 | 1,193.44 | 0.001 | LP387012-0 |
| Calcium \| Serum or Plasma \| Chemistry - non-challenge | 6 | 1,193.44 | 0.001 | LP385966-9 |
| Creatinine \| Serum or Plasma \| Chemistry - non-challenge | 5 | 1,185.06 | 0.001 | LP385359-7 |
| Chloride \| Serum or Plasma \| Chemistry - non-challenge | 5 | 1,180.15 | 0.001 | LP386588-0 |
| Potassium \| Serum or Plasma \| Chemistry - non-challenge | 5 | 1,180.07 | 0.001 | LP386618-5 |
| Bilirubin \| Serum or Plasma \| Chemistry - non-challenge | 5 | 1,178.26 | 0.001 | LP385283-9 |
| Platelets \| Blood \| Hematology and Cell counts | 4 | 1,132.26 | 0.001 | LP393218-5 |
| Hematocrit \| Blood \| Hematology and Cell counts | 4 | 1,120.31 | 0.001 | LP392479-4 |
| Glucose tolerance 2 hours gestational panel \| Urine and Serum or Plasma \| Challenge Bank Panels | 6 | 1,118.05 | 0.001 | LP387239-9 |
| Glucose \| Serum or Plasma \| Chemistry - non-challenge | 4 | 1,117.09 | 0.001 | LP385540-2 |
| Glucose post fasting and meal stimulation panel \| Serum or Plasma \| Challenge Bank Panels | 6 | 1,110.73 | 0.001 | LP419313-4 |
| Lipid and glucose panel \| Serum or Plasma \| Chemistry Panels | 6 | 1,098.64 | 0.001 | LP419298-7 |
| Erythrocyte mean corpuscular volume \| Red Blood Cells \| Hematology and Cell counts | 6 | 1,070.15 | 0.001 | LP393361-3 |
| Sodium \| Serum or Plasma \| Chemistry - non-challenge | 5 | 1,056.01 | 0.001 | LP386648-2 |
| Erythrocyte mean corpuscular hemoglobin concentration \| Red Blood Cells \| Hematology and Cell counts | 6 | 1,044.24 | 0.001 | LP393357-1 |
| Erythrocyte mean corpuscular hemoglobin \| Red Blood Cells \| Hematology and Cell counts | 6 | 1,044.17 | 0.001 | LP393353-0 |
| Leukocytes \| Blood \| Hematology and Cell counts | 4 | 1,032.16 | 0.001 | LP392599-9 |
| Segmented neutrophils \| Blood \| Hematology and Cell counts | 4 | 1,004.50 | 0.001 | LP392658-3 |
| Platelet indices | 4 | 966.85 | 0.001 | LP31668-4 |
| Platelet mean volume \| Blood \| Hematology and Cell counts | 5 | 966.85 | 0.001 | LP393244-1 |
| Protein \| Urine \| Urinalysis | 5 | 949.73 | 0.001 | LP402534-4 |
| Hemoglobin \| Blood \| Hematology and Cell counts | 4 | 932.14 | 0.001 | LP392452-1 |
| Magnesium and phosphate and lactate panel \| Serum or Plasma \| Chemistry Panels | 6 | 888.23 | 0.001 | LP427534-5 |
| Nucleated erythrocytes \| Blood \| Hematology and Cell counts | 4 | 883.78 | 0.001 | LP392558-5 |
| Erythrocytes \| Blood \| Hematology and Cell counts | 4 | 868.24 | 0.001 | LP392503-1 |
| Erythrocyte distribution width \| Red Blood Cells \| Hematology and Cell counts | 6 | 857.74 | 0.001 | LP393348-0 |
| Cell count and Diff panel with Gluc and Prot \| Cerebral spinal fluid \| Hematology and Cell Count Panels | 5 | 789.77 | 0.001 | LP393894-3 |
| Small molecules | 5 | 777.63 | 0.001 | LP31415-0 |
| Nursing physiologic assessment panel \| Patient \| Clinical panels | 4 | 754.52 | 0.001 | LP428885-0 |
| Bilirubin \| Urine \| Urinalysis | 5 | 745.70 | 0.001 | LP402524-5 |
| pH \| Urine \| Urinalysis | 5 | 742.12 | 0.001 | LP402533-6 |
| Ketones \| Urine \| Urinalysis | 5 | 729.47 | 0.001 | LP402528-6 |
| Microbiology CNAMTS panel \| Urine \| Microbiology Panels | 6 | 710.03 | 0.001 | LP380052-3 |
| Nitrite \| Urine \| Urinalysis | 5 | 708.19 | 0.001 | LP402532-8 |
| Genitourinary assessment panel | 4 | 691.41 | 0.001 | 80330-4 |
| Cell count and Differential panel \| Cerebral spinal fluid \| Hematology and Cell Count Panels | 5 | 653.99 | 0.001 | LP393885-1 |
| Specific gravity \| Urine \| Urinalysis | 5 | 641.32 | 0.001 | LP402537-7 |
| Leukocyte esterase \| Urine \| Urinalysis | 5 | 562.81 | 0.001 | LP402530-2 |
| Drug and Toxicology Panels | 4 | 529.24 | 0.001 | LP29683-7 |
| Glucose \| Urine \| Urinalysis | 4 | 492.83 | 0.001 | LP402526-0 |
| Specific antigens | 4 | 432.70 | 0.001 | LP57347-4 |
| Respiratory pathogens RNA 8 panel \| XXX \| Microbiology Panels | 6 | 417.58 | 0.001 | LP380090-3 |
| Influenza virus types A and B and subtypes RNA panel \| XXX \| Microbiology Panels | 6 | 398.45 | 0.001 | LP427045-2 |
| Antibiotic susceptibilities | 4 | 339.94 | 0.001 | LP7755-4 |
| Lipid 1996 panel \| Serum or Plasma \| Chemistry Panels | 6 | 180.13 | 0.001 | LP386973-4 |
| Lipoprofile panel \| Serum or Plasma \| Chemistry Panels | 6 | 175.62 | 0.001 | LP386975-9 |
| Lipid panel with direct LDL \| Serum or Plasma \| Chemistry Panels | 6 | 167.96 | 0.001 | LP386974-2 |
| Lipoprotein metabolism panel \| Serum or Plasma \| Chemistry Panels | 6 | 161.38 | 0.001 | LP386976-7 |
| Fertility Panels | 4 | 126.68 | 0.001 | LP36844-6 |
| Retrograde ejaculation evaluation panel \| Urine \| Fertility Panels | 5 | 126.68 | 0.001 | LP428682-1 |
| Genetic Antimicrobial Resistance | 5 | 103.84 | 0.001 | LP64213-9 |
| Allergy Panels | 4 | 91.42 | 0.001 | LP33267-3 |
| Influenza virus B \| XXX \| Microbiology | 5 | 50.03 | 0.001 | LP378528-6 |
| Herpes virus 6 DNA panel \| XXX \| Microbiology Panels | 6 | 19.72 | 0.001 | LP380002-8 |
| Stone analysis panel \| Calculus (stone) \| Chemistry Panels | 6 | 15.52 | 0.001 | LP386842-1 |
| Coccidioides immitis IgG and IgM panel \| Cerebral spinal fluid \| Microbiology Panels | 6 | 15.39 | 0.001 | LP379937-8 |
| Mycoplasma sp and Ureaplasma sp panel \| XXX \| Microbiology Panels | 6 | 15.39 | 0.001 | LP380069-7 |
| Pancreatic exocrine function panel \| Body fluid \| Challenge Bank Panels | 6 | 13.31 | 0.001 | LP419315-9 |
| Respiratory viral pathogens DNA and RNA panel \| Respiratory specimen \| Microbiology Panels | 6 | 13.13 | 0.001 | LP419284-7 |
| Respiratory pathogens DNA and RNA panel \| Respiratory specimen \| Microbiology Panels | 6 | 13.11 | 0.001 | LP380084-6 |
| Influenza virus A and B and SARS-CoV-2 (COVID-19) and SARS-related CoV RNA panel \| Respiratory specimen \| Microbiology Panels | 6 | 12.31 | 0.001 | LP419290-4 |
| Streptococcus pyogenes \| Throat \| Microbiology | 5 | 11.07 | 0.001 | LP376020-6 |
