# Supplementary material for: A machine learning-based phenotype for long COVID in children: An EHR-based study from the RECOVER program
Source: PLoS One. 2023 Aug 10;18(8):e0289774. doi: 10.1371/journal.pone.0289774 (PMC10414557; doi:10.1371/journal.pone.0289774)
Supplement: S2 Table — These tables show the TreeScan-selected cuts for conditions, labs, procedures, and medications. Each row describes the top node which characterizes the cluster. In other words, the node, together with all descendant codes, defines the feature cluster. (ZIP) [file pone.0289774.s004.zip › Supplementary Table 2c.docx]

| Concept | Tree Level | Log Likelihood Ratio | P value | Procedure code | Vocabulary |
| --- | --- | --- | --- | --- | --- |
| Electrocardiogram, routine ECG with at least 12 leads | 3 | 3,794.95 | 0.001 | 1013012 | CPT4 |
| Echocardiography, transthoracic, real-time with image documentation (2D), includes M-mode recording, when performed, complete, with spectral Doppler echocardiography, and with color flow Doppler echocardiography | 3 | 2,589.93 | 0.001 | 93306 | CPT4 |
| Diagnostic Radiology (Diagnostic Imaging) Procedures | 3 | 2,373.33 | 0.001 | 1010252 | CPT4 |
| Electrocardiogram, routine ECG with at least 12 leads; tracing only, without interpretation and report | 4 | 1,973.67 | 0.001 | 93005 | CPT4 |
| Diagnostic Radiology (Diagnostic Imaging) Procedures of the Chest | 4 | 1,512.90 | 0.001 | 1010334 | CPT4 |
| Doppler echocardiography color flow velocity mapping (List separately in addition to codes for echocardiography) | 3 | 1,170.87 | 0.001 | 93325 | CPT4 |
| Radiologic examination, chest | 5 | 1,137.95 | 0.001 | 1031050 | CPT4 |
| Echocardiography, transthoracic, real-time with image documentation (2D), includes M-mode recording, when performed, follow-up or limited study | 3 | 1,135.75 | 0.001 | 93308 | CPT4 |
| Doppler echocardiography, pulsed wave and/or continuous wave with spectral display (List separately in addition to codes for echocardiographic imaging) | 3 | 1,086.26 | 0.001 | 1013066 | CPT4 |
| Electrocardiogram, routine ECG with at least 12 leads; interpretation and report only | 4 | 1,054.08 | 0.001 | 93010 | CPT4 |
| Doppler echocardiography, pulsed wave and/or continuous wave with spectral display (List separately in addition to codes for echocardiographic imaging); follow-up or limited study (List separately in addition to codes for echocardiographic imaging) | 4 | 1,010.09 | 0.001 | 93321 | CPT4 |
| Myocardial strain imaging using speckle tracking-derived assessment of myocardial mechanics (List separately in addition to codes for echocardiography imaging) | 3 | 850.79 | 0.001 | 93356 | CPT4 |
| Surgical Procedures on the Cardiovascular System | 3 | 814.42 | 0.001 | 1006056 | CPT4 |
| Surgical Procedures on Arteries and Veins | 4 | 807.89 | 0.001 | 1006359 | CPT4 |
| Electrocardiogram, routine ECG with at least 12 leads; with interpretation and report | 4 | 769.13 | 0.001 | 93000 | CPT4 |
| Diagnostic Ultrasound Procedures | 3 | 761.95 | 0.001 | 1010759 | CPT4 |
| Administration, Circulatory, Transfusion | 3 | 747.36 | 0.001 | 302 | ICD10PCS |
| Vascular Introduction and Injection Procedures | 5 | 727.15 | 0.001 | 1013922 | CPT4 |
| Administration @ Circulatory @ Transfusion @ Peripheral Vein | 4 | 724.48 | 0.001 | 3023 | ICD10PCS |
| Administration @ Circulatory @ Transfusion @ Peripheral Vein @ Percutaneous | 5 | 724.48 | 0.001 | 30233 | ICD10PCS |
| Diagnostic Radiology (Diagnostic Imaging) Procedures of the Heart | 4 | 651.59 | 0.001 | 1010594 | CPT4 |
| Radiologic examination, chest; 2 views | 6 | 598.01 | 0.001 | 71046 | CPT4 |
| Collection of venous blood by venipuncture | 7 | 562.84 | 0.001 | 36415 | CPT4 |
| Radiologic examination, chest; single view | 6 | 550.27 | 0.001 | 71045 | CPT4 |
| Diagnostic Ultrasound Procedures of the Abdomen and Retroperitoneum | 4 | 506.45 | 0.001 | 1010774 | CPT4 |
| Venous Procedures | 6 | 488.25 | 0.001 | 1006654 | CPT4 |
| External electrocardiographic recording up to 48 hours by continuous rhythm recording and storage | 3 | 448.27 | 0.001 | 1013030 | CPT4 |
| Ultrasound, abdominal, real time with image documentation | 5 | 440.48 | 0.001 | 1010775 | CPT4 |
| Diagnostic Radiology (Diagnostic Imaging) Procedures of the Abdomen | 4 | 426.45 | 0.001 | 1010520 | CPT4 |
| Diagnostic Radiology (Diagnostic Imaging) Procedures of the Head and Neck | 4 | 337.01 | 0.001 | 1010253 | CPT4 |
| Therapeutic, prophylactic, or diagnostic injection (specify substance or drug) | 3 | 336.20 | 0.001 | 1019333 | CPT4 |
| Bronchodilation responsiveness, spirometry as in 94010, pre- and post-bronchodilator administration | 3 | 317.17 | 0.001 | 94060 | CPT4 |
| Intravenous infusion, for therapy, prophylaxis, or diagnosis (specify substance or drug) | 3 | 280.17 | 0.001 | 1019331 | CPT4 |
| Transfusion Medicine Procedures | 3 | 265.15 | 0.001 | 1012085 | CPT4 |
| Therapeutic procedure, 1 or more areas, each 15 minutes | 3 | 261.45 | 0.001 | 1013511 | CPT4 |
| Physical therapy evaluation: moderate complexity, requiring these components: A history of present problem with 1-2 personal factors and/or comorbidities that impact the plan of care; An examination of body systems using standardized tests and measures in | 3 | 253.29 | 0.001 | 97162 | CPT4 |
| Insertion of Central Venous Access Device | 7 | 243.80 | 0.001 | 1006697 | CPT4 |
| Central Venous Access Procedures | 6 | 237.21 | 0.001 | 1006696 | CPT4 |
| Radiologic examination, abdomen | 5 | 228.88 | 0.001 | 1031051 | CPT4 |
| Ultrasound, abdominal, real time with image documentation; limited (eg, single organ, quadrant, follow-up) | 6 | 228.69 | 0.001 | 76705 | CPT4 |
| Ultrasound, abdominal, real time with image documentation; complete | 6 | 226.47 | 0.001 | 76700 | CPT4 |
| Extracorporeal or Systemic Assistance and Performance, Physiological Systems, Performance | 3 | 212.01 | 0.001 | 5A1 | ICD10PCS |
| Intravenous infusion, for therapy, prophylaxis, or diagnosis (specify substance or drug); initial, up to 1 hour | 4 | 206.36 | 0.001 | 96365 | CPT4 |
| External electrocardiographic recording up to 48 hours by continuous rhythm recording and storage; recording (includes connection, recording, and disconnection) | 4 | 192.74 | 0.001 | 93225 | CPT4 |
| Therapeutic procedure, 1 or more areas, each 15 minutes; therapeutic exercises to develop strength and endurance, range of motion and flexibility | 4 | 189.08 | 0.001 | 97110 | CPT4 |
| Therapeutic, prophylactic, or diagnostic injection (specify substance or drug); each additional sequential intravenous push of a new substance/drug (List separately in addition to code for primary procedure) | 4 | 187.45 | 0.001 | 96375 | CPT4 |
| Therapeutic, prophylactic, or diagnostic injection (specify substance or drug); intravenous push, single or initial substance/drug | 4 | 186.39 | 0.001 | 96374 | CPT4 |
| Therapeutic activities, direct (one-on-one) patient contact (use of dynamic activities to improve functional performance), each 15 minutes | 3 | 183.65 | 0.001 | 97530 | CPT4 |
| Doppler echocardiography, pulsed wave and/or continuous wave with spectral display (List separately in addition to codes for echocardiographic imaging); complete | 4 | 175.28 | 0.001 | 93320 | CPT4 |
| Transthoracic echocardiography for congenital cardiac anomalies | 3 | 165.42 | 0.001 | 1013051 | CPT4 |
| Spirometry, including graphic record, total and timed vital capacity, expiratory flow rate measurement(s), with or without maximal voluntary ventilation | 3 | 162.71 | 0.001 | 94010 | CPT4 |
| Extracorporeal or Systemic Assistance and Performance @ Physiological Systems @ Performance @ Respiratory | 4 | 158.54 | 0.001 | 5A19 | ICD10PCS |
| Duplex scan of extremity veins including responses to compression and other maneuvers | 3 | 152.22 | 0.001 | 1013199 | CPT4 |
| Computed tomographic angiography, chest (noncoronary), with contrast material(s), including noncontrast images, if performed, and image postprocessing | 5 | 149.02 | 0.001 | 71275 | CPT4 |
| Computed tomography, abdomen and pelvis | 5 | 148.87 | 0.001 | 1020544 | CPT4 |
| Heart and Great Vessels, Insertion | 3 | 148.86 | 0.001 | 02H | ICD10PCS |
| Continuous positive airway pressure ventilation (CPAP), initiation and management | 3 | 147.42 | 0.001 | 94660 | CPT4 |
| Occupational therapy evaluation, moderate complexity, requiring these components: An occupational profile and medical and therapy history, which includes an expanded review of medical and/or therapy records and additional review of physical, cognitive, or | 3 | 147.15 | 0.001 | 97166 | CPT4 |
| Radiologic examination, abdomen; 1 view | 6 | 143.48 | 0.001 | 74018 | CPT4 |
| Computed tomography, abdomen and pelvis; with contrast material(s) | 6 | 139.10 | 0.001 | 74177 | CPT4 |
| Self-care/home management training (eg, activities of daily living (ADL) and compensatory training, meal preparation, safety procedures, and instructions in use of assistive technology devices/adaptive equipment) direct one-on-one contact, each 15 minutes | 3 | 138.59 | 0.001 | 97535 | CPT4 |
| Ultrasound guidance for vascular access requiring ultrasound evaluation of potential access sites, documentation of selected vessel patency, concurrent realtime ultrasound visualization of vascular needle entry, with permanent recording and reporting (Lis | 5 | 136.92 | 0.001 | 76937 | CPT4 |
| Ultrasonic Guidance Procedures | 4 | 130.49 | 0.001 | 1010824 | CPT4 |
| Medical and Surgical @ Heart and Great Vessels @ Insertion @ Superior Vena Cava @ Percutaneous @ Infusion Device | 6 | 129.42 | 0.001 | 02HV33 | ICD10PCS |
| Medical and Surgical @ Heart and Great Vessels @ Insertion @ Superior Vena Cava @ Percutaneous | 5 | 129.42 | 0.001 | 02HV3 | ICD10PCS |
| Insertion of Infusion Device into Superior Vena Cava, Percutaneous Approach | 7 | 129.42 | 0.001 | 02HV33Z | ICD10PCS |
| Medical and Surgical @ Heart and Great Vessels @ Insertion @ Superior Vena Cava | 4 | 129.25 | 0.001 | 02HV | ICD10PCS |
| Medical and Surgical @ Respiratory System @ Insertion @ Trachea | 4 | 128.61 | 0.001 | 0BH1 | ICD10PCS |
| Respiratory System, Insertion | 3 | 127.94 | 0.001 | 0BH | ICD10PCS |
| 3D rendering with interpretation and reporting of computed tomography, magnetic resonance imaging, ultrasound, or other tomographic modality with image postprocessing under concurrent supervision | 5 | 127.42 | 0.001 | 1010748 | CPT4 |
| Magnetic resonance (eg, proton) imaging, brain (including brain stem) | 5 | 117.22 | 0.001 | 1010326 | CPT4 |
| Physical therapy evaluation: high complexity, requiring these components: A history of present problem with 3 or more personal factors and/or comorbidities that impact the plan of care; An examination of body systems using standardized tests and measures | 3 | 115.42 | 0.001 | 97163 | CPT4 |
| Administration, Physiological Systems and Anatomical Regions, Introduction | 3 | 114.41 | 0.001 | 3E0 | ICD10PCS |
| Other Diagnostic Radiology (Diagnostic Imaging) Related Procedures | 4 | 109.76 | 0.001 | 1010693 | CPT4 |
| Surgical Procedures on the Respiratory System | 3 | 107.65 | 0.001 | 1005690 | CPT4 |
| Transthoracic echocardiography for congenital cardiac anomalies; complete | 4 | 102.72 | 0.001 | 93303 | CPT4 |
| Arterial catheterization or cannulation for sampling, monitoring or transfusion (separate procedure) | 6 | 99.59 | 0.001 | 1006739 | CPT4 |
| Arterial catheterization or cannulation for sampling, monitoring or transfusion (separate procedure); percutaneous | 7 | 98.65 | 0.001 | 36620 | CPT4 |
| Radiologic examination, chest; single view, frontal | 6 | 97.37 | 0.001 | 71010 | CPT4 |
| Radiologic examination, chest | 5 | 97.37 | 0.001 | 1010335 | CPT4 |
| Arterial Procedures | 5 | 96.01 | 0.001 | 1006737 | CPT4 |
| Duplex scan of arterial inflow and venous outflow of abdominal, pelvic, scrotal contents and/or retroperitoneal organs | 3 | 90.52 | 0.001 | 1013203 | CPT4 |
| Computed tomography, head or brain | 5 | 89.91 | 0.001 | 1010296 | CPT4 |
| Ultrasound, retroperitoneal (eg, renal, aorta, nodes), real time with image documentation | 5 | 88.85 | 0.001 | 1010778 | CPT4 |
| Unlisted pulmonary service or procedure | 3 | 87.63 | 0.001 | 94799 | CPT4 |
| Computed tomography, thorax, diagnostic | 5 | 86.70 | 0.001 | 1036223 | CPT4 |
| Noninvasive ear or pulse oximetry for oxygen saturation; multiple determinations (eg, during exercise) | 4 | 85.11 | 0.001 | 94761 | CPT4 |
| Duplex scan of extremity veins including responses to compression and other maneuvers; unilateral or limited study | 4 | 83.39 | 0.001 | 93971 | CPT4 |
| Administration @ Circulatory @ Transfusion @ Central Vein @ Percutaneous | 5 | 79.08 | 0.001 | 30243 | ICD10PCS |
| Administration @ Circulatory @ Transfusion @ Central Vein | 4 | 79.08 | 0.001 | 3024 | ICD10PCS |
| Insertion of peripherally inserted central venous catheter (PICC), without subcutaneous port or pump, including all imaging guidance, image documentation, and all associated radiological supervision and interpretation required to perform the insertion | 8 | 78.90 | 0.001 | 1035169 | CPT4 |
| Surgical Procedures on the Larynx | 4 | 78.75 | 0.001 | 1005814 | CPT4 |
| Radiologic examination, abdomen; 2 views | 6 | 77.52 | 0.001 | 74019 | CPT4 |
| Ultrasound, soft tissues of head and neck (eg, thyroid, parathyroid, parotid), real time with image documentation | 5 | 77.43 | 0.001 | 76536 | CPT4 |
| Extracorporeal or Systemic Assistance and Performance, Physiological Systems, Assistance | 3 | 76.98 | 0.001 | 5A0 | ICD10PCS |
| Monitoring | 3 | 76.33 | 0.001 | 1035629 | CPT4 |
| Diagnostic Radiology (Diagnostic Imaging) Procedures of the Spine and Pelvis | 4 | 76.20 | 0.001 | 1010367 | CPT4 |
| Extracorporeal or Systemic Assistance and Performance @ Physiological Systems @ Assistance @ Respiratory | 4 | 74.32 | 0.001 | 5A09 | ICD10PCS |
| Magnetic resonance (eg, proton) imaging, brain (including brain stem); without contrast material, followed by contrast material(s) and further sequences | 6 | 74.11 | 0.001 | 70553 | CPT4 |
| Gastrointestinal System, Excision | 3 | 73.84 | 0.001 | 0DB | ICD10PCS |
| Manipulation chest wall, such as cupping, percussing, and vibration to facilitate lung function | 3 | 73.67 | 0.001 | 1013246 | CPT4 |
| Duplex scan of arterial inflow and venous outflow of abdominal, pelvic, scrotal contents and/or retroperitoneal organs; complete study | 4 | 72.80 | 0.001 | 93975 | CPT4 |
| Ultrasound, retroperitoneal (eg, renal, aorta, nodes), real time with image documentation; complete | 6 | 72.36 | 0.001 | 76770 | CPT4 |
| Measurement and Monitoring, Physiological Systems, Monitoring | 3 | 71.37 | 0.001 | 4A1 | ICD10PCS |
| Physical therapy evaluation: low complexity, requiring these components: A history with no personal factors and/or comorbidities that impact the plan of care; An examination of body system(s) using standardized tests and measures addressing 1-2 elements f | 3 | 71.23 | 0.001 | 97161 | CPT4 |
| Computed tomography, head or brain; without contrast material | 6 | 68.13 | 0.001 | 70450 | CPT4 |
| Radiologic examination, chest, 2 views, frontal and lateral | 5 | 67.03 | 0.001 | 1014245 | CPT4 |
| Radiologic examination, chest, 2 views, frontal and lateral | 6 | 67.03 | 0.001 | 71020 | CPT4 |
| Transthoracic echocardiography for congenital cardiac anomalies; follow-up or limited study | 4 | 64.62 | 0.001 | 93304 | CPT4 |
| Occupational therapy evaluation, low complexity, requiring these components: An occupational profile and medical and therapy history, which includes a brief history including review of medical and/or therapy records relating to the presenting problem; An | 3 | 64.09 | 0.001 | 97165 | CPT4 |
| Medical and Surgical @ Central Nervous System and Cranial Nerves @ Drainage @ Spinal Canal @ Percutaneous | 5 | 63.62 | 0.001 | 009U3 | ICD10PCS |
| Medical and Surgical @ Central Nervous System and Cranial Nerves @ Drainage @ Spinal Canal | 4 | 63.49 | 0.001 | 009U | ICD10PCS |
| Medical and Surgical @ Central Nervous System and Cranial Nerves @ Drainage @ Spinal Canal @ Percutaneous @ No Device | 6 | 62.38 | 0.001 | 009U3Z | ICD10PCS |
| Diagnostic Ultrasound Procedures of the Head and Neck | 4 | 62.32 | 0.001 | 1010760 | CPT4 |
| Drainage of Spinal Canal, Percutaneous Approach, Diagnostic | 7 | 57.60 | 0.001 | 009U3ZX | ICD10PCS |
| Central Nervous System and Cranial Nerves, Drainage | 3 | 52.94 | 0.001 | 009 | ICD10PCS |
| Respiratory System, Drainage | 3 | 52.77 | 0.001 | 0B9 | ICD10PCS |
| Computed tomography, thorax, diagnostic; with contrast material(s) | 6 | 50.36 | 0.001 | 71260 | CPT4 |
| Noninvasive ear or pulse oximetry for oxygen saturation; by continuous overnight monitoring (separate procedure) | 4 | 46.73 | 0.001 | 94762 | CPT4 |
| Magnetic resonance (eg, proton) imaging, brain (including brain stem); without contrast material | 6 | 45.41 | 0.001 | 70551 | CPT4 |
| Occupational therapy evaluation, high complexity, requiring these components: An occupational profile and medical and therapy history, which includes review of medical and/or therapy records and extensive additional review of physical, cognitive, or psych | 3 | 44.02 | 0.001 | 97167 | CPT4 |
| Administration @ Physiological Systems and Anatomical Regions @ Introduction @ Central Vein @ Percutaneous | 5 | 41.21 | 0.001 | 3E043 | ICD10PCS |
| Administration @ Physiological Systems and Anatomical Regions @ Introduction @ Central Vein | 4 | 41.21 | 0.001 | 3E04 | ICD10PCS |
| Intravenous infusion, for therapy, prophylaxis, or diagnosis (specify substance or drug); additional sequential infusion of a new drug/substance, up to 1 hour (List separately in addition to code for primary procedure) | 4 | 39.81 | 0.001 | 96367 | CPT4 |
| Manipulation chest wall, such as cupping, percussing, and vibration to facilitate lung function; subsequent | 4 | 38.57 | 0.001 | 94668 | CPT4 |
| Pressurized or nonpressurized inhalation treatment for acute airway obstruction for therapeutic purposes and/or for diagnostic purposes such as sputum induction with an aerosol generator, nebulizer, metered dose inhaler or intermittent positive pressure b | 3 | 38.30 | 0.001 | 94640 | CPT4 |
| Noninvasive ear or pulse oximetry for oxygen saturation | 3 | 38.28 | 0.001 | 1013256 | CPT4 |
| Presumptive Drug Class Screening Procedures | 4 | 37.55 | 0.001 | 1021850 | CPT4 |
| Drug test(s), presumptive, any number of drug classes, any number of devices or procedures | 5 | 37.55 | 0.001 | 1035668 | CPT4 |
| Ultrasound, extremity, nonvascular, real-time with image documentation | 5 | 35.94 | 0.001 | 1020555 | CPT4 |
| Drug Assay Procedures | 3 | 35.54 | 0.001 | 1021848 | CPT4 |
| Manipulation chest wall, such as cupping, percussing, and vibration to facilitate lung function; initial demonstration and/or evaluation | 4 | 35.21 | 0.001 | 94667 | CPT4 |
| Magnetic resonance (eg, proton) imaging, spinal canal and contents, without contrast material, followed by contrast material(s) and further sequences | 5 | 32.47 | 0.001 | 1010408 | CPT4 |
| Intravenous infusion, for therapy, prophylaxis, or diagnosis (specify substance or drug); each additional hour (List separately in addition to code for primary procedure) | 4 | 31.93 | 0.001 | 96366 | CPT4 |
| Other Central Venous Access Procedures | 7 | 30.53 | 0.001 | 1018484 | CPT4 |
| Radiologic examination, abdomen | 5 | 29.82 | 0.001 | 1010521 | CPT4 |
| Health behavior intervention, individual, face-to-face; initial 30 minutes | 3 | 29.53 | 0.001 | 96158 | CPT4 |
| Long-term EEG Setup | 3 | 29.24 | 0.001 | 1035628 | CPT4 |
| Electroencephalogram (EEG) continuous recording, with video when performed, setup, patient education, and takedown when performed, administered in person by EEG technologist, minimum of 8 channels | 4 | 29.24 | 0.001 | 95700 | CPT4 |
| Surgical Procedures on the Trachea and Bronchi | 4 | 28.57 | 0.001 | 1005883 | CPT4 |
| Electroencephalogram with video (VEEG), review of data, technical description by EEG technologist, each increment of 12-26 hours | 4 | 27.71 | 0.001 | 1035679 | CPT4 |
| Injection, Drainage, or Aspiration Procedures on the Spine and Spinal Cord | 5 | 25.20 | 0.001 | 1009341 | CPT4 |
| Esophagogastroduodenoscopy, flexible, transoral | 7 | 25.13 | 0.001 | 1007260 | CPT4 |
| Esophagogastroduodenoscopy Procedures | 6 | 25.13 | 0.001 | 1021431 | CPT4 |
| Diagnostic Ultrasound Procedures of the Extremities | 4 | 22.91 | 0.001 | 1010819 | CPT4 |
| Surgical Procedures on the Digestive System | 3 | 22.28 | 0.001 | 1006964 | CPT4 |
| Endoscopy Procedures on the Esophagus | 5 | 22.18 | 0.001 | 1007241 | CPT4 |
| Surgical Procedures on the Esophagus | 4 | 22.15 | 0.001 | 1007214 | CPT4 |
| Diagnostic Radiology (Diagnostic Imaging) Procedures of the Gastrointestinal Tract | 4 | 21.23 | 0.001 | 1010537 | CPT4 |
| Magnetic resonance (eg, proton) imaging, abdomen | 5 | 20.94 | 0.001 | 1010531 | CPT4 |
| Surgical Procedures on the Spine and Spinal Cord | 4 | 20.79 | 0.001 | 1009340 | CPT4 |
| Endoscopy Procedures on the Trachea and Bronchi | 5 | 18.99 | 0.001 | 1005896 | CPT4 |
| Demonstration and/or evaluation of patient utilization of an aerosol generator, nebulizer, metered dose inhaler or IPPB device | 3 | 18.47 | 0.001 | 94664 | CPT4 |
| Bronchoscopy, rigid or flexible, including fluoroscopic guidance, when performed | 6 | 17.17 | 0.001 | 1005899 | CPT4 |
| Diagnostic Ultrasound Procedures of the Pelvis Non-Obstetrical | 5 | 16.52 | 0.001 | 1010809 | CPT4 |
| Surgical Procedures on the Nervous System | 3 | 15.70 | 0.001 | 1009068 | CPT4 |
| Radiologic examination | 5 | 15.56 | 0.001 | 1010289 | CPT4 |
| Polysomnography | 3 | 15.47 | 0.001 | 1013314 | CPT4 |
| Endoscopy Procedures on the Larynx | 5 | 15.37 | 0.001 | 1005837 | CPT4 |
| Esophagogastroduodenoscopy, flexible, transoral; with biopsy, single or multiple | 8 | 14.33 | 0.001 | 43239 | CPT4 |
| Diagnostic Ultrasound Procedures of the Pelvis | 4 | 13.79 | 0.001 | 1010784 | CPT4 |
| Therapeutic procedure, 1 or more areas, each 15 minutes; neuromuscular reeducation of movement, balance, coordination, kinesthetic sense, posture, and/or proprioception for sitting and/or standing activities | 4 | 13.41 | 0.001 | 97112 | CPT4 |
| Radiologic examination; neck, soft tissue | 6 | 12.85 | 0.001 | 70360 | CPT4 |
| Diagnostic Nuclear Medicine Procedures | 4 | 11.68 | 0.001 | 1010938 | CPT4 |
| Nuclear Medicine Procedures | 3 | 11.45 | 0.001 | 1010937 | CPT4 |
| Psychotherapy, 60 minutes with patient | 3 | 9.62 | 0.001 | 90837 | CPT4 |
| Psychological and Neuropsychological Test Administration and Scoring | 3 | 8.34 | 0.003 | 1035140 | CPT4 |
