# Supplementary material for: A machine learning-based phenotype for long COVID in children: An EHR-based study from the RECOVER program
Source: PLoS One. 2023 Aug 10;18(8):e0289774. doi: 10.1371/journal.pone.0289774 (PMC10414557; doi:10.1371/journal.pone.0289774)
Supplement: S2 Table — These tables show the TreeScan-selected cuts for conditions, labs, procedures, and medications. Each row describes the top node which characterizes the cluster. In other words, the node, together with all descendant codes, defines the feature cluster. (ZIP) [file pone.0289774.s004.zip › Supplementary Table 2d.docx]

| Concept | Tree Level | Log Likelihood Ratio | P value | Medication code | Vocabulary |
| --- | --- | --- | --- | --- | --- |
| Injectable Product | 1 | 10,916.05 | 0.001 | 1151126 | RxNorm |
| Oral Product | 1 | 5,113.74 | 0.001 | 1151131 | RxNorm |
| Pill | 1 | 4,795.41 | 0.001 | 1151133 | RxNorm |
| sodium chloride | 1 | 2,396.33 | 0.001 | 9863 | RxNorm |
| sodium chloride 9 MG/ML | 2 | 2,230.15 | 0.001 | 1661411 | RxNorm |
| glucose | 1 | 1,699.06 | 0.001 | 4850 | RxNorm |
| glucose 50 MG/ML | 2 | 1,662.66 | 0.001 | 315789 | RxNorm |
| Chewable Product | 1 | 1,373.91 | 0.001 | 1294716 | RxNorm |
| famotidine | 1 | 1,302.36 | 0.001 | 4278 | RxNorm |
| sodium chloride Injectable Product | 2 | 1,266.59 | 0.001 | 1159317 | RxNorm |
| prednisone Oral Product | 2 | 1,261.11 | 0.001 | 1161705 | RxNorm |
| prednisone | 1 | 1,261.11 | 0.001 | 8640 | RxNorm |
| prednisone Oral Tablet | 2 | 1,243.87 | 0.001 | 373585 | RxNorm |
| prednisone Pill | 2 | 1,243.34 | 0.001 | 1161706 | RxNorm |
| potassium chloride | 1 | 1,064.73 | 0.001 | 8591 | RxNorm |
| famotidine Oral Product | 2 | 867.11 | 0.001 | 1159021 | RxNorm |
| ceftriaxone | 1 | 849.17 | 0.001 | 2193 | RxNorm |
| diphenhydramine | 1 | 843.84 | 0.001 | 3498 | RxNorm |
| ceftriaxone Injectable Product | 2 | 839.64 | 0.001 | 1152108 | RxNorm |
| Oral Liquid Product | 1 | 779.94 | 0.001 | 1151137 | RxNorm |
| acetaminophen | 1 | 734.02 | 0.001 | 161 | RxNorm |
| sodium chloride Injectable Solution | 2 | 706.56 | 0.001 | 373901 | RxNorm |
| sodium chloride 9 MG/ML Injectable Solution | 3 | 678.94 | 0.001 | 313002 | RxNorm |
| glucose / sodium chloride Injectable Product | 2 | 528.06 | 0.001 | 1165817 | RxNorm |
| glucose / sodium chloride Injection | 2 | 527.95 | 0.001 | 1794561 | RxNorm |
| glucose Injectable Product | 2 | 520.50 | 0.001 | 1165819 | RxNorm |
| heparin | 1 | 512.81 | 0.001 | 5224 | RxNorm |
| 500 ML glucose 50 MG/ML / sodium chloride 9 MG/ML Injection | 3 | 510.97 | 0.001 | 1795344 | RxNorm |
| 1000 ML glucose 50 MG/ML / potassium chloride 0.02 MEQ/ML / sodium chloride 9 MG/ML Injection | 3 | 475.02 | 0.001 | 615107 | RxNorm |
| acetaminophen Pill | 2 | 469.10 | 0.001 | 1152843 | RxNorm |
| acetaminophen Oral Product | 2 | 434.79 | 0.001 | 1152842 | RxNorm |
| potassium chloride 0.02 MEQ/ML | 2 | 434.15 | 0.001 | 316538 | RxNorm |
| heparin Injectable Product | 2 | 421.66 | 0.001 | 1856274 | RxNorm |
| prednisolone | 1 | 419.51 | 0.001 | 8638 | RxNorm |
| prednisolone Oral Product | 2 | 413.26 | 0.001 | 1165759 | RxNorm |
| prednisolone Oral Solution | 2 | 407.38 | 0.001 | 373575 | RxNorm |
| prednisolone Oral Liquid Product | 2 | 407.38 | 0.001 | 1165758 | RxNorm |
| prednisolone 3 MG/ML | 2 | 406.92 | 0.001 | 317474 | RxNorm |
| glucose / potassium chloride / sodium chloride Injectable Product | 2 | 405.75 | 0.001 | 1165813 | RxNorm |
| glucose / potassium chloride / sodium chloride Injection | 2 | 405.75 | 0.001 | 1863604 | RxNorm |
| acetaminophen Oral Tablet | 2 | 394.97 | 0.001 | 369097 | RxNorm |
| famotidine 8 MG/ML | 2 | 387.39 | 0.001 | 315922 | RxNorm |
| famotidine Oral Suspension | 2 | 387.39 | 0.001 | 372139 | RxNorm |
| famotidine Oral Liquid Product | 2 | 387.39 | 0.001 | 1159020 | RxNorm |
| lidocaine | 1 | 382.20 | 0.001 | 6387 | RxNorm |
| Inhalant Product | 1 | 372.00 | 0.001 | 1151123 | RxNorm |
| sodium chloride Injection | 2 | 354.21 | 0.001 | 1807545 | RxNorm |
| prednisolone 3 MG/ML Oral Solution | 3 | 330.63 | 0.001 | 283077 | RxNorm |
| epinephrine 1 MG/ML | 2 | 327.61 | 0.001 | 328316 | RxNorm |
| calcium chloride | 1 | 319.49 | 0.001 | 1901 | RxNorm |
| epinephrine Injectable Product | 2 | 314.68 | 0.001 | 1163887 | RxNorm |
| acetaminophen Injectable Product | 2 | 312.25 | 0.001 | 1152840 | RxNorm |
| acetaminophen Injection | 2 | 312.25 | 0.001 | 1803956 | RxNorm |
| acetaminophen 10 MG/ML | 2 | 311.89 | 0.001 | 483015 | RxNorm |
| omeprazole | 1 | 306.31 | 0.001 | 7646 | RxNorm |
| ondansetron | 1 | 304.38 | 0.001 | 26225 | RxNorm |
| acetaminophen 325 MG | 2 | 294.33 | 0.001 | 315263 | RxNorm |
| diphenhydramine Oral Product | 2 | 293.86 | 0.001 | 1158449 | RxNorm |
| 100 ML acetaminophen 10 MG/ML Injection | 3 | 270.55 | 0.001 | 483017 | RxNorm |
| lactate | 1 | 249.91 | 0.001 | 114202 | RxNorm |
| potassium chloride 0.004 MEQ/ML | 2 | 249.28 | 0.001 | 665002 | RxNorm |
| sodium chloride 0.103 MEQ/ML | 2 | 248.87 | 0.001 | 797835 | RxNorm |
| sodium lactate 0.028 MEQ/ML | 2 | 248.87 | 0.001 | 797836 | RxNorm |
| epinephrine | 1 | 245.48 | 0.001 | 3992 | RxNorm |
| acetaminophen 325 MG Oral Tablet | 3 | 243.33 | 0.001 | 313782 | RxNorm |
| lidocaine Topical Product | 2 | 243.21 | 0.001 | 1164666 | RxNorm |
| lidocaine Topical Cream | 2 | 213.65 | 0.001 | 377740 | RxNorm |
| Oral Powder Product | 1 | 211.16 | 0.001 | 1294711 | RxNorm |
| midazolam Injectable Product | 2 | 208.67 | 0.001 | 1164715 | RxNorm |
| polyethylene glycol 3350 | 1 | 202.91 | 0.001 | 221147 | RxNorm |
| polyethylene glycol 3350 17000 MG | 2 | 200.59 | 0.001 | 1870356 | RxNorm |
| Disintegrating Oral Product | 1 | 199.96 | 0.001 | 1294713 | RxNorm |
| ondansetron Pill | 2 | 183.40 | 0.001 | 1161034 | RxNorm |
| ondansetron Oral Product | 2 | 183.18 | 0.001 | 1161033 | RxNorm |
| ibuprofen Oral Product | 2 | 180.36 | 0.001 | 1156277 | RxNorm |
| ibuprofen | 1 | 180.17 | 0.001 | 5640 | RxNorm |
| polyethylene glycol 3350 Oral Product | 2 | 179.79 | 0.001 | 1162922 | RxNorm |
| polyethylene glycol 3350 Powder for Oral Solution | 2 | 179.79 | 0.001 | 1870358 | RxNorm |
| polyethylene glycol 3350 Oral Powder Product | 2 | 179.66 | 0.001 | 1870357 | RxNorm |
| ibuprofen Pill | 2 | 175.77 | 0.001 | 1156278 | RxNorm |
| ibuprofen Oral Tablet | 2 | 163.80 | 0.001 | 370674 | RxNorm |
| polyethylene glycol 3350 17000 MG Powder for Oral Solution | 3 | 163.04 | 0.001 | 876193 | RxNorm |
| ondansetron Disintegrating Oral Tablet | 2 | 155.01 | 0.001 | 373149 | RxNorm |
| ondansetron Disintegrating Oral Product | 2 | 155.01 | 0.001 | 1295332 | RxNorm |
| ondansetron 4 MG | 2 | 152.81 | 0.001 | 328450 | RxNorm |
| Topical Product | 1 | 142.27 | 0.001 | 1151122 | RxNorm |
| midazolam Injectable Solution | 2 | 139.34 | 0.001 | 379133 | RxNorm |
| midazolam | 1 | 132.69 | 0.001 | 6960 | RxNorm |
| lorazepam | 1 | 131.74 | 0.001 | 6470 | RxNorm |
| albuterol Inhalant Product | 2 | 127.00 | 0.001 | 1154602 | RxNorm |
| albuterol | 1 | 126.16 | 0.001 | 435 | RxNorm |
| ondansetron 2 MG/ML | 2 | 124.90 | 0.001 | 328448 | RxNorm |
| ondansetron Injectable Product | 2 | 122.93 | 0.001 | 1161031 | RxNorm |
| midazolam 1 MG/ML | 2 | 121.61 | 0.001 | 328485 | RxNorm |
| Rectal Product | 1 | 120.48 | 0.001 | 1151127 | RxNorm |
| fentanyl | 1 | 118.40 | 0.001 | 4337 | RxNorm |
| acetaminophen 32 MG/ML | 2 | 118.06 | 0.001 | 315262 | RxNorm |
| acetaminophen Oral Liquid Product | 2 | 117.50 | 0.001 | 1152841 | RxNorm |
| lidocaine Injectable Product | 2 | 113.03 | 0.001 | 1164658 | RxNorm |
| rocuronium bromide 10 MG/ML | 2 | 109.70 | 0.001 | 998229 | RxNorm |
| rocuronium Injectable Solution | 2 | 109.65 | 0.001 | 375623 | RxNorm |
| rocuronium Injectable Product | 2 | 109.65 | 0.001 | 1156444 | RxNorm |
| rocuronium | 1 | 109.65 | 0.001 | 68139 | RxNorm |
| ondansetron 4 MG Disintegrating Oral Tablet | 3 | 104.55 | 0.001 | 104894 | RxNorm |
| ondansetron Injectable Solution | 2 | 100.33 | 0.001 | 376327 | RxNorm |
| sennosides, USP | 1 | 100.28 | 0.001 | 36387 | RxNorm |
| sennosides, USP Oral Product | 2 | 98.28 | 0.001 | 1159068 | RxNorm |
| fentanyl Injectable Product | 2 | 92.32 | 0.001 | 1159056 | RxNorm |
| ondansetron 2 MG/ML Injectable Solution | 3 | 87.47 | 0.001 | 283504 | RxNorm |
| acetaminophen Oral Suspension | 2 | 87.21 | 0.001 | 370509 | RxNorm |
| calcium chloride 0.0014 MEQ/ML | 2 | 85.26 | 0.001 | 847624 | RxNorm |
| fluticasone | 1 | 85.07 | 0.001 | 41126 | RxNorm |
| calcium chloride / lactate / potassium chloride / sodium chloride Injectable Product | 2 | 84.96 | 0.001 | 1154438 | RxNorm |
| calcium chloride 0.0014 MEQ/ML / potassium chloride 0.004 MEQ/ML / sodium chloride 0.103 MEQ/ML / sodium lactate 0.028 MEQ/ML Injectable Solution | 3 | 84.96 | 0.001 | 847630 | RxNorm |
| calcium chloride / lactate / potassium chloride / sodium chloride Injectable Solution | 2 | 84.96 | 0.001 | 876077 | RxNorm |
| dexmedetomidine | 1 | 84.34 | 0.001 | 48937 | RxNorm |
| ketorolac | 1 | 80.66 | 0.001 | 35827 | RxNorm |
| ibuprofen 200 MG | 2 | 79.36 | 0.001 | 316074 | RxNorm |
| albuterol Metered Dose Inhaler | 2 | 77.36 | 0.001 | 745678 | RxNorm |
| albuterol 0.09 MG/ACTUAT | 2 | 76.95 | 0.001 | 329498 | RxNorm |
| lidocaine Injectable Solution | 2 | 76.37 | 0.001 | 372599 | RxNorm |
| ketorolac Injectable Product | 2 | 76.26 | 0.001 | 1160966 | RxNorm |
| lidocaine hydrochloride 10 MG/ML | 2 | 75.96 | 0.001 | 1010032 | RxNorm |
| dexmedetomidine Injectable Product | 2 | 64.47 | 0.001 | 1154546 | RxNorm |
| ibuprofen 200 MG Oral Tablet | 3 | 62.30 | 0.001 | 310965 | RxNorm |
| influenza A virus (H1N1) antigen / influenza A virus (H3N2) antigen / influenza B virus antigen Prefilled Syringe | 2 | 61.33 | 0.001 | 1657137 | RxNorm |
| influenza B virus B/Phuket/3073/2013 antigen 0.03 MG/ML | 2 | 60.84 | 0.001 | 1657229 | RxNorm |
| influenza A virus (H1N1) antigen / influenza A virus (H3N2) antigen / influenza B virus antigen Injectable Product | 2 | 60.71 | 0.001 | 1657136 | RxNorm |
| influenza B virus antigen | 1 | 60.15 | 0.001 | 1657134 | RxNorm |
| influenza A virus (H3N2) antigen | 1 | 60.15 | 0.001 | 1657131 | RxNorm |
| influenza A virus (H1N1) antigen | 1 | 60.15 | 0.001 | 1657128 | RxNorm |
| acetaminophen 32 MG/ML Oral Suspension | 3 | 59.29 | 0.001 | 307668 | RxNorm |
| fentanyl Injection | 2 | 58.10 | 0.001 | 1735002 | RxNorm |
| fentanyl 0.05 MG/ML | 2 | 57.00 | 0.001 | 328264 | RxNorm |
| ibuprofen 20 MG/ML | 2 | 53.61 | 0.001 | 316073 | RxNorm |
| ibuprofen Oral Liquid Product | 2 | 52.97 | 0.001 | 1156276 | RxNorm |
| ibuprofen Oral Suspension | 2 | 52.97 | 0.001 | 370672 | RxNorm |
| dexmedetomidine Injection | 2 | 52.66 | 0.001 | 1718899 | RxNorm |
| albuterol Inhalation Solution | 2 | 52.33 | 0.001 | 2108226 | RxNorm |
| fluticasone Inhalant Product | 2 | 47.47 | 0.001 | 1165655 | RxNorm |
| 2 ML fentanyl 0.05 MG/ML Injection | 3 | 46.91 | 0.001 | 1735003 | RxNorm |
| cholecalciferol Oral Product | 2 | 43.61 | 0.001 | 1156133 | RxNorm |
| cholecalciferol | 1 | 43.48 | 0.001 | 2418 | RxNorm |
| ibuprofen 20 MG/ML Oral Suspension | 3 | 39.04 | 0.001 | 197803 | RxNorm |
| fluticasone Metered Dose Inhaler | 2 | 38.10 | 0.001 | 746403 | RxNorm |
| Zofran Oral Product | 2 | 36.57 | 0.001 | 1188160 | RxNorm |
| ketorolac tromethamine 30 MG/ML | 2 | 34.15 | 0.001 | 860095 | RxNorm |
| Zofran Pill | 2 | 31.83 | 0.001 | 1188161 | RxNorm |
| morphine | 1 | 31.52 | 0.001 | 7052 | RxNorm |
| acetaminophen Oral Solution | 2 | 30.30 | 0.001 | 370506 | RxNorm |
| Tylenol Oral Product | 2 | 29.87 | 0.001 | 1187311 | RxNorm |
| acetaminophen Oral Suspension [Tylenol] | 2 | 29.82 | 0.001 | 828554 | RxNorm |
| acetaminophen 32 MG/ML Oral Suspension [Tylenol] | 3 | 29.82 | 0.001 | 828555 | RxNorm |
| acetaminophen 32 MG/ML [Tylenol] | 2 | 29.82 | 0.001 | 828553 | RxNorm |
| Tylenol Oral Liquid Product | 2 | 29.82 | 0.001 | 1187310 | RxNorm |
| acetaminophen 32 MG/ML Oral Solution | 3 | 29.70 | 0.001 | 307675 | RxNorm |
| NDA020983 60 ACTUAT albuterol 0.09 MG/ACTUAT Metered Dose Inhaler | 3 | 29.39 | 0.001 | 801092 | RxNorm |
| cetirizine Pill | 2 | 29.36 | 0.001 | 1152447 | RxNorm |
| cetirizine Oral Tablet | 2 | 25.93 | 0.001 | 371364 | RxNorm |
| sodium chloride Inhalant Product | 2 | 25.54 | 0.001 | 1159316 | RxNorm |
| sodium chloride 4.5 MG/ML | 2 | 25.49 | 0.001 | 1794564 | RxNorm |
| Nasal Product | 1 | 23.19 | 0.001 | 1151130 | RxNorm |
| Flovent Inhalant Product | 2 | 23.13 | 0.001 | 1169793 | RxNorm |
| morphine Injectable Product | 2 | 23.08 | 0.001 | 1156360 | RxNorm |
| fluticasone Metered Dose Inhaler [Flovent] | 2 | 22.63 | 0.001 | 746412 | RxNorm |
| dexmedetomidine 0.004 MG/ML | 2 | 22.08 | 0.001 | 1249680 | RxNorm |
| hydrocortisone | 1 | 21.04 | 0.001 | 5492 | RxNorm |
| ibuprofen 20 MG/ML [Junifen] | 2 | 14.82 | 0.001 | 564197 | RxNorm |
| Junifen Oral Liquid Product | 2 | 14.82 | 0.001 | 1167873 | RxNorm |
| ibuprofen Oral Suspension [Junifen] | 2 | 14.82 | 0.001 | 365908 | RxNorm |
| ibuprofen 20 MG/ML Oral Suspension [Junifen] | 3 | 14.82 | 0.001 | 105850 | RxNorm |
| Junifen Oral Product | 2 | 14.82 | 0.001 | 1167874 | RxNorm |
| Ophthalmic Product | 1 | 13.85 | 0.001 | 1151135 | RxNorm |
| morphine sulfate 2 MG/ML | 2 | 13.58 | 0.001 | 892588 | RxNorm |
| propofol | 1 | 12.37 | 0.001 | 8782 | RxNorm |
| clavulanate | 1 | 11.74 | 0.001 | 48203 | RxNorm |
| amoxicillin / clavulanate Oral Product | 2 | 11.74 | 0.001 | 1152874 | RxNorm |
| albuterol 0.83 MG/ML | 2 | 11.72 | 0.002 | 346188 | RxNorm |
| fluticasone Nasal Product | 2 | 11.24 | 0.002 | 1165656 | RxNorm |
| fluticasone Metered Dose Nasal Spray | 2 | 11.24 | 0.002 | 1797889 | RxNorm |
| oxycodone Oral Product | 2 | 10.54 | 0.003 | 1161682 | RxNorm |
| fluticasone propionate 0.044 MG/ACTUAT | 2 | 10.51 | 0.003 | 895993 | RxNorm |
| cetirizine Oral Product | 2 | 10.44 | 0.003 | 1152446 | RxNorm |
| oxycodone | 1 | 10.44 | 0.003 | 7804 | RxNorm |
| fluticasone propionate 0.05 MG/ACTUAT | 2 | 10.40 | 0.003 | 896014 | RxNorm |
| cetirizine | 1 | 10.37 | 0.003 | 20610 | RxNorm |
| epinephrine Auto-Injector | 2 | 10.22 | 0.003 | 1661387 | RxNorm |
| propofol Injectable Product | 2 | 10.21 | 0.003 | 1162323 | RxNorm |
| propofol 10 MG/ML | 2 | 9.38 | 0.006 | 345859 | RxNorm |
